# The schedule of ATR inhibitor AZD6738 can potentiate or abolish antitumor immune responses to radiotherapy

**DOI:** 10.1172/jci.insight.165615

**Published:** 2023-02-22

**Authors:** Frank P. Vendetti, Pinakin Pandya, David A. Clump, Sandra Schamus-Haynes, Meysam Tavakoli, Maria diMayorca, Naveed M. Islam, Jina Chang, Greg M. Delgoffe, Jan H. Beumer, Christopher J. Bakkenist

**Affiliations:** 1 Department of Radiation Oncology, UPMC Hillman Cancer Center, School of Medicine, University of Pittsburgh, Pittsburgh, Pennsylvania, USA.; 2 Department of Radiation Oncology, School of Medicine, West Virginia University, Morgantown, West Virginia, USA.; 3 Department of Immunology and; 4 Department of Medicine, UPMC Hillman Cancer Center, School of Medicine, University of Pittsburgh, Pittsburgh, Pennsylvania, USA.; 5 Department of Pharmaceutical Sciences, School of Pharmacy, University of Pittsburgh, Pittsburgh, Pennsylvania, USA.; 6 Division of Hematology-Oncology, Department of Medicine, and; 7 Department of Pharmacology and Chemical Biology, UPMC Hillman Cancer Center, School of Medicine, University of Pittsburgh, Pittsburgh, Pennsylvania, USA.

**Keywords:** Immunology, Oncology, Cancer immunotherapy, Radiation therapy, T cells

## Abstract

Inhibitors of the DNA damage signaling kinase ATR increase tumor cell killing by chemotherapies that target DNA replication forks but also kill rapidly proliferating immune cells including activated T cells. Nevertheless, ATR inhibitor (ATRi) and radiotherapy (RT) can be combined to generate CD8^+^ T cell–dependent antitumor responses in mouse models. To determine the optimal schedule of ATRi and RT, we determined the impact of short-course versus prolonged daily treatment with AZD6738 (ATRi) on responses to RT (days 1–2). Short-course ATRi (days 1–3) plus RT caused expansion of tumor antigen–specific, effector CD8^+^ T cells in the tumor-draining lymph node (DLN) at 1 week after RT. This was preceded by acute decreases in proliferating tumor-infiltrating and peripheral T cells and a rapid proliferative rebound after ATRi cessation, increased inflammatory signaling (IFN-β, chemokines, particularly CXCL10) in tumors, and an accumulation of inflammatory cells in the DLN. In contrast, prolonged ATRi (days 1–9) prevented the expansion of tumor antigen–specific, effector CD8^+^ T cells in the DLN, and entirely abolished the therapeutic benefit of short-course ATRi with RT and anti–PD-L1. Our data argue that ATRi cessation is essential to allow CD8^+^ T cell responses to both RT and immune checkpoint inhibitors.

## Introduction

Most chemotherapies target DNA replication forks, and their efficacy is associated with both the direct killing of proliferating tumor cells and the stimulation of antitumor immune responses that is often associated with transient leukocytopenia followed by a rebound proliferation of immune cell populations (1, 2). A major clinical goal is to maximize the direct killing of tumor cells, while concurrently potentiating the rebound proliferation of tumor-specific effector cells. The DNA damage response (DDR) is a signaling system that integrates DNA metabolism with the cell cycle (3). DDR kinase inhibitors (DDRis) potentiate tumor cell killing by DNA-damaging therapies (2, 4). Studies showing that DDRis generate innate immune responses have generated interest in combining DDRis with immunotherapy (5–7). However, how DDRis directly impact immune cells has not been systematically investigated.

ATR is a DDR kinase activated at damaged replication forks and resected DNA double-strand breaks. Six ATR inhibitors (ATRis) have advanced to phase I and phase II clinical trials: ceralasertib (AZD6738), berzosertib (M6620, VX-970), elimusertib (BAY 1895344), RP-3500, M4344 (VX-803), and M1774 (8–13). Substantial preclinical evidence demonstrates that AZD6738 (ATRi) sensitizes cells to DNA-damaging agents (14–24). Preclinical studies also show that ATRi can potentiate antitumor immune responses, but the underlying mechanisms are not fully understood (17, 19, 25–28). Phase I data show that ATRi suppresses circulating monocytes and proliferating T cells, and that neutropenia and thrombocytopenia are dose- and schedule-limiting toxicities, which indicates that ATRi may limit CD8^+^ T cell–dependent responses (29–31). Nevertheless, patients are being treated with immunotherapy and ATRi twice daily for up to 2 weeks (ClinicalTrials.gov).

We previously demonstrated that ATRi combines with conformal tumor radiotherapy (RT) to generate durable, CD8^+^ T cell–dependent antitumor responses in murine cancer models (17). We reported that short-course, 3-day treatment with ATRi transiently reduced both proliferating and effector CD8^+^ T cell populations in spleens and tumor-infiltrating lymphocytes (TILs) of tumor-bearing mice. We did not address either the kinetics of T cell recovery after ATRi treatment or the impact of ATRi on T cell priming and activation in the tumor-draining lymph node (DLN). Here we investigate the effects of short-course and prolonged, daily ATRi treatment schedules on T cell responses in TILs and the DLN of tumor-bearing mice. We show that ATRi integrates with RT to generate antigen-specific CD8^+^ T cell responses in the periphery, but only after ATRi is removed. We show that prolonged daily ATRi treatment abolishes CD8^+^ T cell responses after RT in both the CT26 and B16 syngeneic tumor models.

## Results

### Short-course ATRi integrates with RT to generate an expansion of tumor antigen–specific CD8^+^ T cells in the periphery.

Short-course AZD6738 treatment on days 1–3 (ATRi QDx3) combined with RT on days 1–2 (RT) increased the infiltration of functional CD8^+^ T cells in CT26 tumors by 10 days after RT, and this correlated with antitumor response (17). Preclinical data demonstrate that CD8^+^ T cells in the DLN are required for the efficacy of PD-1/PD-L1 checkpoint blockade (32). Therefore, we hypothesized that activation and expansion of tumor-reactive, effector CD8^+^ T cells in the DLN after ATRi QDx3 plus RT contributed to the antitumor responses observed.

**Figure 1:**
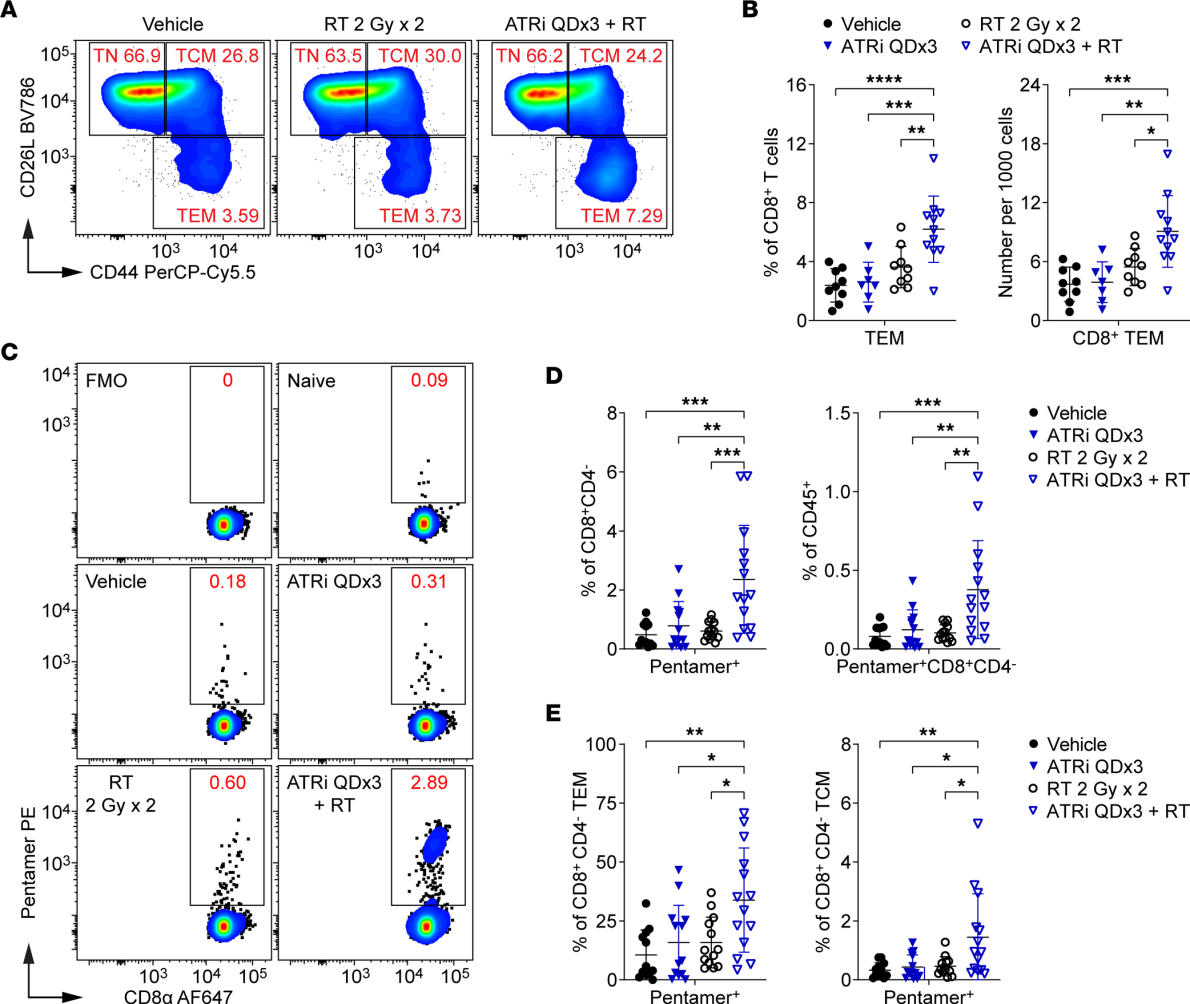
Short-course ATRi plus RT promotes tumor antigen–specific CD8^+^ T cell expansion in the DLN. (**A**–**E**) CT26 tumor–bearing mice were treated with ATRi on days 1–3 (ATRi QDx3), RT on days 1–2 (RT 2 Gy x 2), ATRi QDx3 + RT, or vehicle, and tumor-draining lymph nodes (DLNs) were immunoprofiled at day 9. (**A**) Representative cytograms depicting CD62L and CD44 expression on CD8^+^ T cells. Activated and naive CD8^+^ T cell subsets were defined as effector/effector memory (Tem; CD44^hi^CD62L^lo^), central memory (Tcm; CD62L^hi^CD44^hi^), or naive (Tn; CD62L^hi^CD44^lo^). (**B**) Quantitation of CD8^+^ Tem cells as percentages of CD8^+^ T cells or per 1,000 cells stained. Data from at least 4 independent experiments with 1–3 mice per group. *n* = 9 Vehicle, 7 ATRi QDx3, 9 RT, 11 ATRi QDx3 + RT. (**C**–**E**) Tumor antigen–specific CD8^+^ T cells were labeled with AH1 Pentamer. (**C**) Representative cytograms depicting Pentamer^+^ CD8^+^ T cells. Fluorescence-minus-one (no Pentamer) and naive (negative, no tumor) controls shown. (**D**) Quantitation of Pentamer^+^ CD8^+^ T cells as percentages of CD8^+^CD4^–^ cells or CD45^+^ immune cells. (**E**) Quantitation of Pentamer^+^ CD8^+^ Tem and Tcm cells as percentages of CD8^+^CD4^–^ Tem and Tcm cells. (**D** and **E**) Data from at least 5 independent experiments with 1–5 mice per group. *n* = 12 Vehicle, 13 ATRi QDx3, 13 RT, 14 ATRi QDx3 + RT. (**B**, **D**, and **E**) Mean and SD bars shown. *P < 0.05, **P < 0.01, ***P < 0.001, ****P < 0.0001 by ANOVA with Tukey’s multiple-comparison test.

We examined activated effector/effector memory (Tem; CD44^hi^CD62L^lo^) and central memory (Tcm; CD62L^hi^CD44^hi^) CD8^+^ T cell subsets, as well as the naive (Tn; CD62L^hi^CD44^lo^) CD8^+^ T cell subset, in the DLN at day 9 (Figure 1A). ATRi QDx3 plus RT increased the proportion of CD8^+^ Tem cells in the DLN compared with all other treatments (Figure 1B). This correlated with a reduction in the Tn subset, but no change in the Tcm subset, in the DLN of ATRi QDx3 plus RT–treated mice (Supplemental Figure 1A; supplemental material available online with this article; https://doi.org/10.1172/jci.insight.165615DS1). While ATRi QDx3 plus RT did not increase the total number of CD8^+^ T cells in the DLN at day 9 (Supplemental Figure 1B), CD8^+^ Tem cells constituted a greater number of the DLN cells stained (Figure 1B). Therefore, ATRi QDx3 plus RT generates an expansion of effector/effector memory (Tem) CD8^+^ T cells in the DLN by day 9.

To determine whether this CD8^+^ Tem cell expansion in the DLN was tumor antigen specific, we labeled CD8^+^ T cells with an MHC class I H-2Ld–restricted Pentamer loaded with the immunodominant CT26 antigen, the AH1 peptide (SPSYVYHQF) of the murine leukemia virus gp70 envelope protein (33–35) (Figure 1C). While there were no differences among treatments in the number of CD8^+^CD4^–^ cells in the DLN at day 9 (Supplemental Figure 1C), ATRi QDx3 plus RT increased Pentamer-positive CD8^+^CD4^–^ cells, both as percentages of CD8^+^CD4^–^ cells and of CD45^+^ immune cells, compared with all other treatments (Figure 1D). Pentamer-positive cells in both the CD8^+^ Tem and Tcm subsets also increased after ATRi QDx3 plus RT compared with all other treatment groups (Figure 1E). Notably, Pentamer-positive cells constituted a large portion of the Tem cell population and, in some ATRi QDx3 plus RT–treated mice, constituted more than half of the Tem cell pool (Figure 1E). These data show that short-course ATRi plus RT generates an expansion of tumor antigen–specific, effector/effector memory CD8^+^ T cells in the DLN by day 9, one week after RT.

### Proliferating T cells are depleted by short-course ATRi but rapidly rebound after cessation of ATRi.

**Figure 2:**
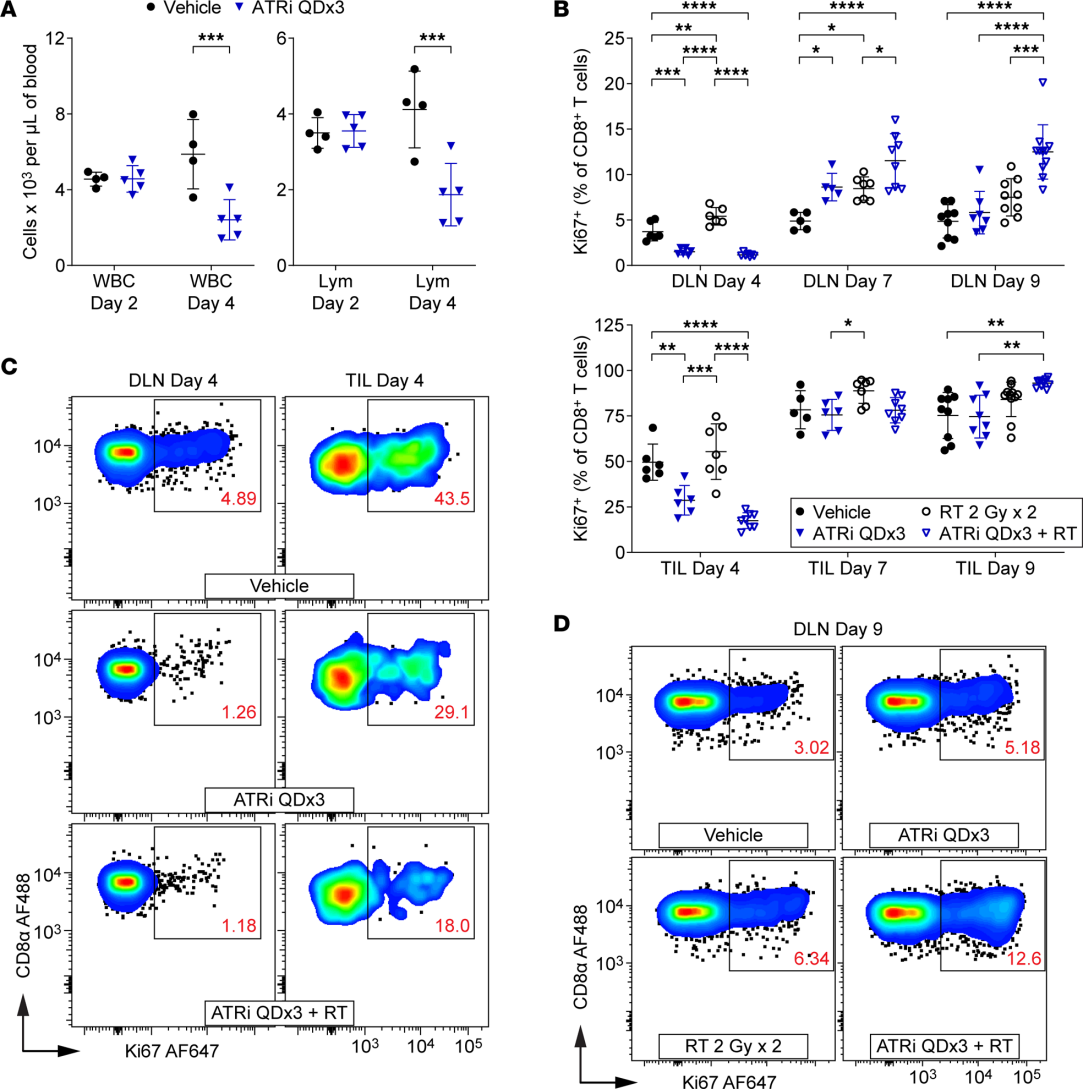
Proliferating T cells are depleted by short-course ATRi but rapidly rebound upon cessation of ATRi treatment. (**A**) BALB/c mice were treated with ATRi QDx3 or vehicle. Complete blood counts (CBCs) were performed at days 2 (24 hours after the first dose of ATRi) and 4 (24 hours after the third dose of ATRi). *n* = 4 vehicle, 5 ATRi QDx3. Quantitation of white blood cells (WBC) and lymphocytes (Lym) per microliter of blood, with mean and SD bars, is shown. ***P < 0.001 by 2-way ANOVA with Šidák’s multiple-comparison test. (**B**–**D**) CT26 tumor–bearing mice were treated with ATRi QDx3, RT 2 Gy x 2, ATRi QDx3 + RT, or vehicle. DLN and TILs were immunoprofiled at days 4, 7, and 9. (**B**) Quantitation of proliferating (Ki67^+^) CD8^+^ T cells, as percentages of CD8^+^ T cells, in DLN and TILs at days 4, 7, and 9. Data from at least 3 (day 4), 2 (day 7), or 4 (day 9) independent experiments with 1–4 mice per group. *n* at days 4/7/9 = 6/5/9 Vehicle, 6/6(5 DLN)/8(7 DLN) ATRi QDx3, 7(6 DLN)/7/11(9 DLN) RT, 8(7 DLN)/8/11 ATRi QDx3 + RT. Mean and SD bars shown. *P < 0.05, **P < 0.01, ***P < 0.001, ****P < 0.0001 by ANOVA with Tukey’s multiple-comparison test. (**C** and **D**) Representative cytograms depicting Ki67 expression in CD8^+^ T cells in DLN and TILs at day 4 (**C**) and TILs at day 9 (**D**).

Since ATRi QDx3 transiently reduced proliferating T cell populations in spleens and TILs of CT26 tumor–bearing mice (17), we investigated the kinetics of T cell recovery in the DLN and TILs following ATRi cessation. We first examined the impact of ATRi on immune populations in BALB/c mice treated with 75 mg/kg AZD6738 on days 1–3 (ATRi QDx3) by performing complete blood counts at day 2 (24 hours after the first dose of ATRi) and day 4 (24 hours after the third dose of ATRi). One dose of ATRi did not reduce white blood cell (WBC), lymphocyte, or neutrophil counts by day 2, but 3 doses of ATRi reduced counts of all three by day 4 (Figure 2A and Supplemental Figure 2A). One dose of ATRi did, however, reduce monocytes by day 2 (Supplemental Figure 2A). These data are consistent with the effects of ATRi on WBCs in patients (29–31).

Next, we examined the impact of ATRi QDx3, alone and in combination with RT on days 1–2 (RT 2 Gy x 2), in T cell populations in CT26 tumor–bearing mice. We immunoprofiled T cell populations in the DLN and TILs at day 4, the time at which we observed decreases in circulating lymphocytes, at day 7, and at day 9, the time at which we observed expansion of tumor antigen–specific CD8^+^ T cells. Consistent with our prior findings in spleens and TILs (17), ATRi QDx3 reduced proliferating CD8^+^ T cells (Figure 2, B and C), proliferating conventional CD4^+^ T cells (CD4^+^ Tconvs) (Supplemental Figure 2B), and proliferating regulatory T cells (Tregs) (Supplemental Figure 2C) in the DLN and TILs at day 4. On average, ATRi QDx3 plus RT further decreased Ki67^+^ CD8^+^ T cells in TILs compared with ATRi QDx3 alone, but this difference was not statistically significant (Figure 2B). Consistent with these findings, we recently showed that ATRi directly kills rapidly proliferating, but not naive, CD8^+^ T cells ex vivo (36).

Upon cessation of ATRi treatment, all T cell populations exhibited a rapid proliferative rebound in the DLN by day 7, with ATRi QDx3 and ATRi QDx3 plus RT increasing the percentages of proliferating CD8^+^ T cells (Figure 2B). Similar rebounds were observed in CD4^+^ Tconvs and Tregs in the DLN (Supplemental Figure 2, B and C). Proliferating CD8^+^ T cells in TILs of mice treated with ATRi QDx3, with or without RT, recovered to near vehicle control percentages by day 7 (Figure 2B). The proliferative rebound in CD4^+^ Tconv and Treg TILs of ATRi QDx3–treated mice, independent of RT, was evident by day 7 and persisted to day 9 (Supplemental Figure 2, B and C). However, continued proliferation of CD8^+^ T cells in TILs at day 9 occurred only in ATRi QDx3 plus RT–treated mice (Figure 2B). Moreover, at day 9, proliferating CD8^+^ T cells were increased in the DLN of ATRi QDx3 plus RT–treated mice compared with all other treatment groups (Figure 2, B and D). Conversely, proliferating CD4^+^ Tconvs and Tregs were increased relative to vehicle control but not RT alone (Supplemental Figure 2, B and C). These data demonstrate a proliferative rebound in T cells within 4 days of cessation of short-course ATRi treatment, and that the persistent proliferative response in CD8^+^ T cells at day 9 in the DLN and TILs requires both ATRi QDx3 and RT.

### Repopulation of the CD8^+^ T cell tumor infiltrate after short-course ATRi plus RT requires transit of CD8^+^ T cells from the periphery.

**Figure 3:**
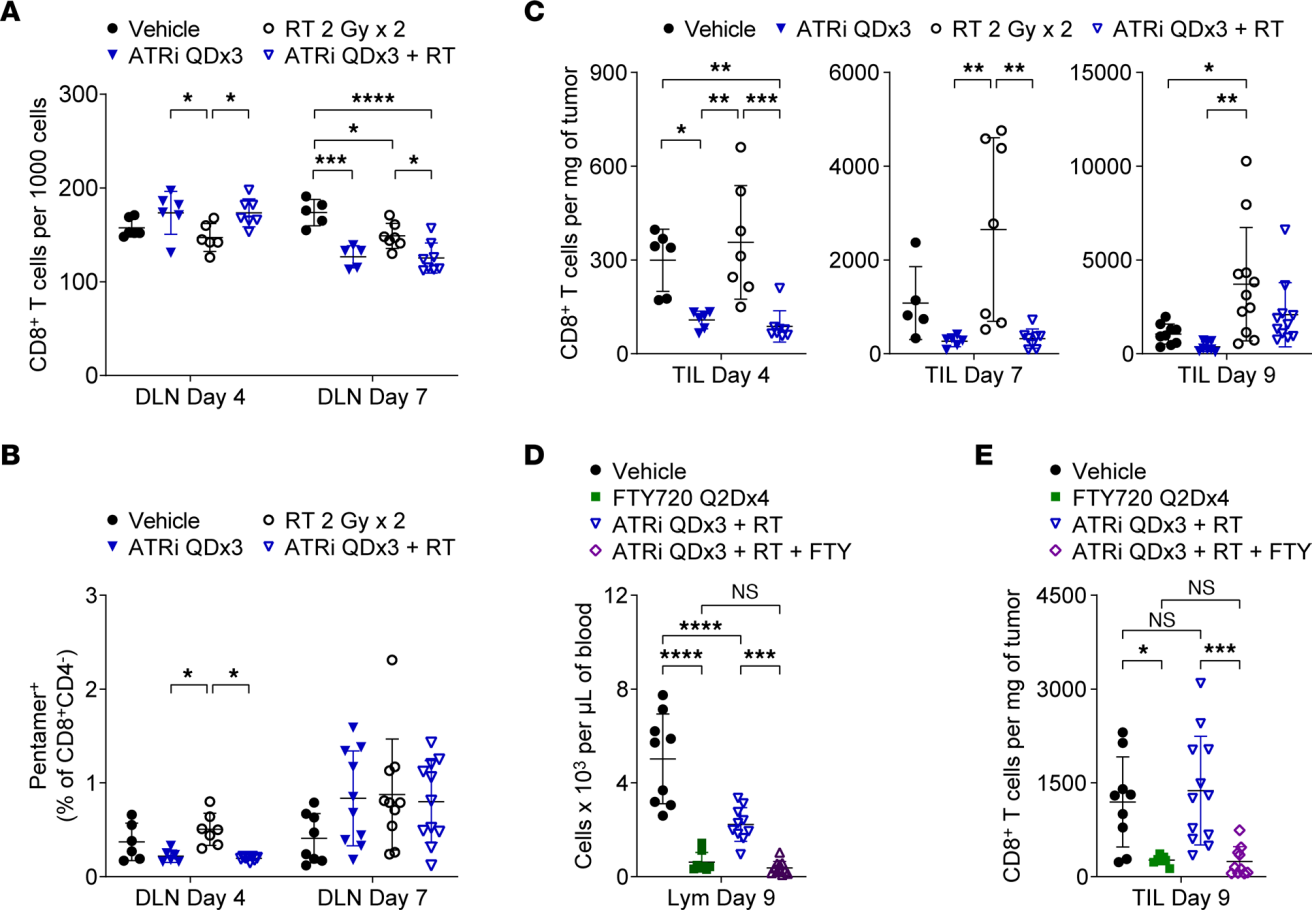
CD8^+^ T cell recovery in TILs after short-course ATRi plus RT requires transit of CD8^+^ T cells from the periphery. (**A**–**C**) CT26 tumor–bearing mice treated with ATRi QDx3, RT 2 Gy x 2, ATRi QDx3 + RT, or vehicle. (**A**) Quantitation of CD8^+^ T cells, per 1,000 cells stained, in DLN at days 4 and 7. (**B**) Quantitation of Pentamer^+^ CD8^+^ T cells, as percentages of CD8^+^CD4^–^ cells, in DLN at days 4 and 7. Data from at least 3 experiments per time point with 1–5 mice per group. *n* at days 4/7 = 6/8 Vehicle, 6/10 ATRi QDx3, 7/10 RT, 8/11 ATRi QDx3 + RT. (**C**) Quantitation of CD8^+^ T cells, per milligram of tumor, in the TILs at days 4, 7, and 9. (**A** and **C**) Data from at least 3 (day 4), 2 (day 7), or 4 (day 9) independent experiments with 1–4 mice per group. *n* at day 4/7/9 = 6/5/9 Vehicle, 6/6(5 DLN)/8 ATRi QDx3, 7(6 DLN)/7/11 RT, 8(7 DLN)/8/11 ATRi QDx3 + RT. (**D** and **E**) CT26 tumor–bearing mice treated with ATRi QDx3 + RT or vehicle, with or without FTY720 Q2Dx4. (**D**) Lymphocyte (Lym) quantitation, per microliter of blood, from CBC at day 9. Data from 3 experiments with 2–4 mice per group. *n* = 9 Vehicle, 8 FTY720 Q2Dx4, 10 ATRi QDx3 + RT, 11 ATRi QDx3 + RT + FTY. (**E**) Quantitation of CD8^+^ T cells, per milligram of tumor, in TILs at day 9. Data from 4 experiments with 1–4 mice per group. (**A**–**E**) Mean and SD bars shown. *P < 0.05, **P < 0.01, ***P < 0.001, ****P < 0.0001 by ANOVA with Tukey’s (**A**–**C**) or Šidák’s (**D** and **E**) multiple-comparison tests.

ATRi QDx3 and ATRi QDx3 plus RT reduced the percentages of proliferating CD8^+^ T cells in the DLN at day 4, but not total CD8^+^ T cells in the DLN (Figure 3A). However, ATRi QDx3, but not ATRi QDx3 plus RT, reduced total CD4^+^ Tconvs and Tregs (Supplemental Figure 3, A and B). Since our quantitation of each T cell population is relative to the total number of DLN cells stained, and not an absolute quantitation, the impact of ATRi on a specific T cell population may be obscured if other immune cells present in the DLN are simultaneously reduced by ATRi treatment. Furthermore, our Ki67^+^ data suggest that the impact of ATRi in CD8^+^ T cells, CD4^+^ Tconvs, and Tregs may differ in magnitude and/or occur with different kinetics. Finally, the proliferating T cell pools constitute unequal and, in the case of CD8^+^ T cells and CD4^+^ Tconvs, small fractions of the total T cell pools in the DLN. That ATRi QDx3 plus RT reduced CD8^+^ Tem and Tcm cells and concomitantly increased the CD8^+^ Tn cell pool in the DLN at day 4 (Supplemental Figure 3C) further demonstrates the acute impact of ATRi treatment on CD8^+^ T cells, consistent with our previous finding in spleens at day 5 (17).

ATRi QDx3 and ATRi QDx3 plus RT reduced CD8^+^ T cells and CD4^+^ Tconvs in the DLN at day 7 (Figure 3A and Supplemental Figure 3A), despite the proliferative rebound in T cell populations observed at this time point. This increased proliferation is likely an effort to recover T cell pools as seen with lymphopenia-induced proliferation (37, 38). Consistent with this premise, at day 9, neither ATRi QDx3 nor ATRi QDx3 plus RT impacted the total number of each T cell population in the DLN (CD8^+^ shown previously in Supplemental Figure 1B and Supplemental Figure 3, A and B).

We also examined tumor antigen–specific CD8^+^ T cells in the DLN at day 4 and day 7. ATRi QDx3 and ATRi QDx3 plus RT reduced Pentamer^+^ CD8^+^ T cells at day 4 compared with RT alone (Figure 3B). At day 7, we observed small, but not statistically significant, increases in Pentamer^+^ CD8^+^ T cells with all treatments (Figure 3B), indicating that ATRi QDx3 plus RT generated a tumor antigen–specific CD8^+^ T cell expansion at day 9 that is caused by neither rebound proliferation following ATRi QDx3 nor a simple delay in antigen-specific CD8^+^ T cell expansion triggered by RT alone at an earlier time point.

In the TILs, ATRi QDx3 and ATRi QDx3 plus RT reduced all three T cell populations by day 4, and none of these T cell populations fully recovered to vehicle control numbers by day 7 (Figure 3C and Supplemental Figure 3, A and B). However, at day 9, we observed repopulation of the TILs in ATRi QDx3 plus RT–treated mice, and tumor-infiltrating CD8^+^ T cells accumulated to at least vehicle control numbers (Figure 3C). ATRi QDx3 plus RT generated an accumulation of CD8^+^ T cells in the TILs that may be associated with in situ proliferation of resident CD8^+^ T cells or infiltration of new CD8^+^ T cells from the periphery. To examine this, we used FTY720 to block egress from the lymphoid tissues (39). One dose of 1 mg/kg FTY720 essentially eliminated T cells and CD8^+^ T cells from circulation for at least 48 hours (Supplemental Figure 4, A and B). Administration of FTY720 on days 1, 3, 5, and 7 (Q2Dx4) essentially eliminated lymphocytes from circulation until day 9 (Figure 3D). Importantly, FTY720 Q2Dx4 reduced WBCs but not monocytes or neutrophils (Supplemental Figure 4C). Therefore, this FTY720 dosing schedule sequestered lymphocytes as expected. We treated mice with ATRi QDx3 plus RT or vehicle, with or without FTY720 Q2Dx4, and immunoprofiled TILs at day 9. Both vehicle-treated and ATRi QDx3 plus RT–treated mice that received FTY720 Q2Dx4 exhibited dramatically reduced CD8^+^ T cell infiltrates compared with the vehicle-treated and ATRi QDx3 plus RT–treated mice that did not receive FTY720 (Figure 3E). These data demonstrate the following: that CD8^+^ T cells from the periphery continue to infiltrate tumors of vehicle-treated mice from day 1 to day 9; that in situ proliferation of tumor-infiltrating CD8^+^ T cells that survive ATRi QDx3 plus RT treatment is not sufficient to account for the accumulated CD8^+^ T cell infiltrate by day 9; and that the observed accumulation of CD8^+^ T cells in TILs of ATRi QDx3 plus RT–treated mice by day 9 requires transit of CD8^+^ T cells from the periphery.

### Short-course ATRi treatment potentiates RT-induced inflammatory cytokines and chemokines in the tumor microenvironment.

Recent studies show that ATRi increases type I interferon (IFN) signaling and the production of proinflammatory cytokines and chemokines after radiation in vitro and in vivo (19, 26). Similar proinflammatory signaling occurs after DNA damage by combined ATR and WEE1 inhibition (28). Combined ATRi and RT is also associated with increased antigen presentation by tumor cells and myeloid cell infiltration into tumors in vivo (19). These data suggest that proinflammatory cytokine signaling following ATRi plus RT may induce priming of the adaptive antitumor immune response.

Using multiplex immunoassays, we examined type I IFN (IFN-α, IFN-β), type II IFN (IFN-γ), proinflammatory cytokine (GM-CSF, IL-6, IL-12p70), and proinflammatory chemokine (CXCL10 [IP-10], CCL2 [MCP-1], CCL3 [MIP-1α], CCL5 [RANTES]) signaling in the tumor microenvironment (TME). IFN-β, IL-6, CXCL10, CCL2, and CCL5 have been reported to be induced in tumor cells by ATRi plus RT in vivo and/or in vitro (19, 26). GM-CSF regulates the maturation and functional activity of monocytes, macrophages, and dendritic cells (DCs) (40). IL-12p70 promotes NK and T cell–mediated IFN-γ production and cytotoxicity (41).

**Figure 4:**
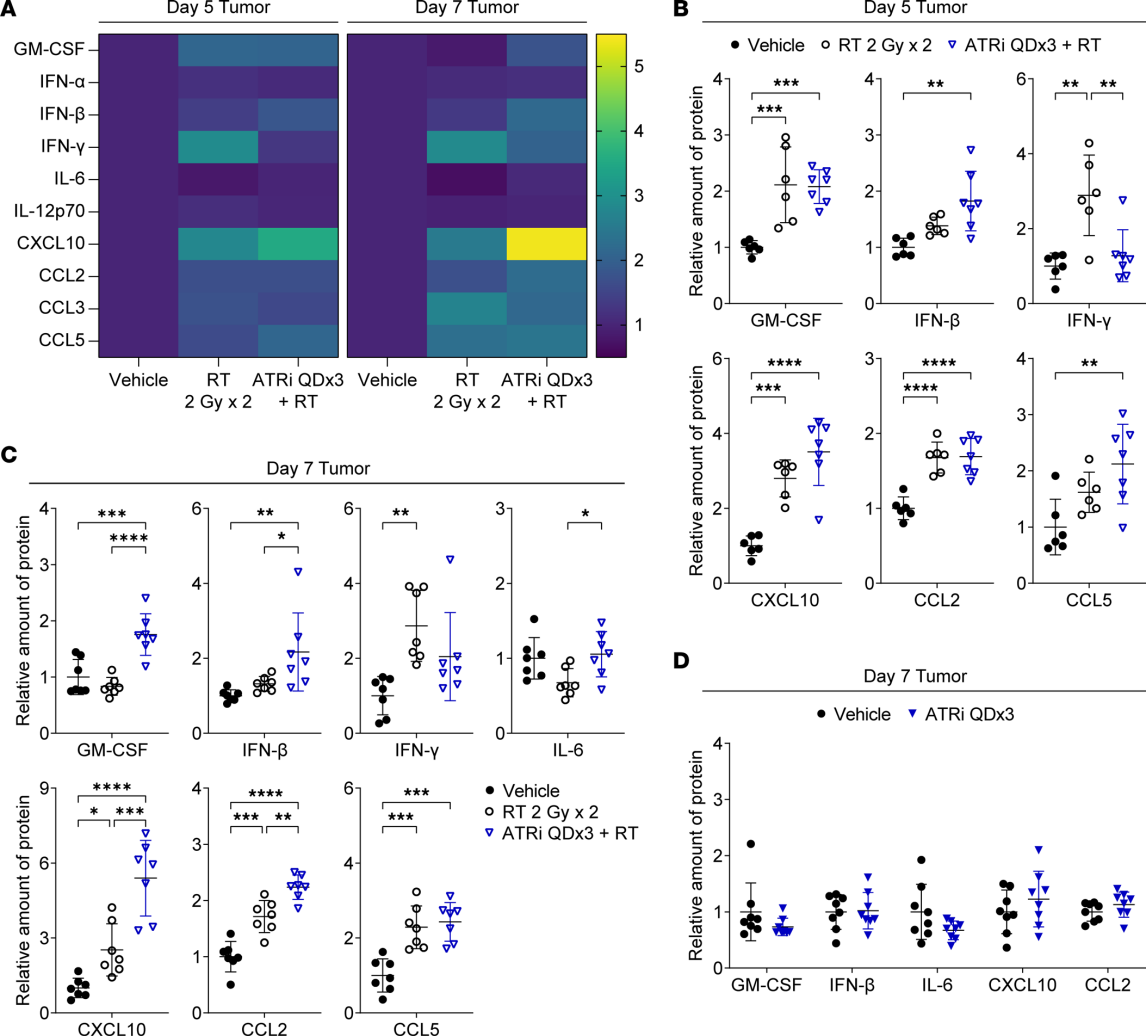
Short-course ATRi treatment potentiates RT-induced inflammatory cytokines and chemokines in the tumor microenvironment. (**A**–**C**) CT26 tumor–bearing mice were treated with RT 2 Gy x 2, ATRi QDx3 + RT, or vehicle. (**A**) Heatmaps depicting the mean relative amount (normalized to vehicle control) of 10 inflammatory cytokines and chemokines in tumors at days 5 and 7. (**B** and **C**) Quantitation of the relative amount of protein (normalized to vehicle control) for a subset of inflammatory cytokines and chemokines in tumors at day 5 (**B**) and day 7 (**C**). Day 5 data from 1 experiment with *n* = 6 Vehicle, 6 RT, 7 ATRi QDx3 + RT. Day 7 data from 2 independent experiments, each with 3–4 mice per group, with total *n* = 7 Vehicle, 7 RT, 7 ATRi QDx3 + RT. (**D**) CT26 tumor–bearing mice were treated with ATRi QDx3 or vehicle, and the relative amounts of protein (normalized to vehicle control) for a subset of inflammatory cytokines and chemokines in tumors at day 7 were quantified. Data from 3 independent experiments, each with 2–3 mice per group. *n* = 8 mice per group. (**B**–**D**) Mean and SD bars shown. (**B** and **C**) *P < 0.05, **P < 0.01, ***P < 0.001, ****P < 0.0001 by ANOVA with Tukey’s multiple-comparison test. (**D**) No significant changes by unpaired, 2-tailed *t* test.

We treated mice with RT, ATRi QDx3 plus RT, or vehicle, and measured the protein levels of cytokines/chemokines in extracts from tumors harvested at day 5 and day 7 (Figure 4A). At day 5, RT alone and ATRi QDx3 plus RT increased levels of GM-CSF, CXCL10, CCL2, and CCL3 compared with vehicle, but ATRi QDx3 did not potentiate levels of these proteins beyond RT alone (Figure 4B and Supplemental Figure 5A). RT alone increased IFN-γ in tumors, while ATRi QDx3 attenuated RT-induced IFN-γ (Figure 4B), which is entirely consistent with our prior finding that ATRi QDx3 attenuated RT-induced increases in tumor-infiltrating, IFN-γ–competent CD8^+^ T cells at this time point (17). While ATRi QDx3 plus RT increased IFN-β and CCL5 compared with vehicle control, the increases were not significant compared with RT alone (Figure 4B). No changes in IFN-α, IL-6, or IL-12p70 were observed at day 5 (Supplemental Figure 5A).

At day 7, ATRi QDx3 plus RT increased GM-CSF and IFN-β relative to both RT alone and vehicle control and potentiated RT-induced increases in CXCL10 and CCL2 (Figure 4C). Both RT alone and ATRi QDx3 plus RT increased CCL5, but no potentiation by ATRi QDx3 over RT alone was observed (Figure 4C). Although not to a statistically significant degree. ATRi QDx3, on average, attenuated RT-induced IFN-γ in tumors at day 7 (Figure 4C). ATRi QDx3 plus RT increased proliferating CD8^+^ T cells in TILs at day 7, but the absolute number of CD8^+^ T cells remained lower than in TILs of RT-treated mice, which likely explains the reduced IFN-γ in ATRi QDx3 plus RT–treated tumors. ATRi QDx3 plus RT did not alter levels of IFN-α, IL-6, IL-12p70, or CCL3 at day 7 (Figure 4C and Supplemental Figure 5B). Therefore, short-course ATRi potentiates RT-induced increases in IFN-β, GM-CSF, CXCL10, and CCL2 in the TME, consistent with previous reports (19, 26).

In our studies, ATRi QDx3 alone did not increase peripheral expansion of CD8^+^ Tem cells or tumor antigen–specific CD8^+^ T cells. To confirm that ATRi QDx3 alone also does not induce proinflammatory cytokines/chemokines in CT26, we treated separate cohorts of mice with ATRi QDx3 or vehicle and assayed GM-CSF, IFN-β, IL-6, CXCL10, CCL2, and CCL3 in tumors at day 7. ATRi QDx3 alone did not increase any of these cytokines or chemokines (Figure 4D and Supplemental Figure 5C). Therefore, our data show that only ATRi QDx3 plus RT results in both potentiation of proinflammatory cytokines/chemokines in the tumor to day 7 and promotion of tumor antigen–specific CD8^+^ T cell expansion in the DLN at day 9.

### Short-course ATRi plus RT promotes accumulation of inflammation-associated innate immune cells and increased CD8^+^ T cell activation in the DLN.

**Figure 5:**
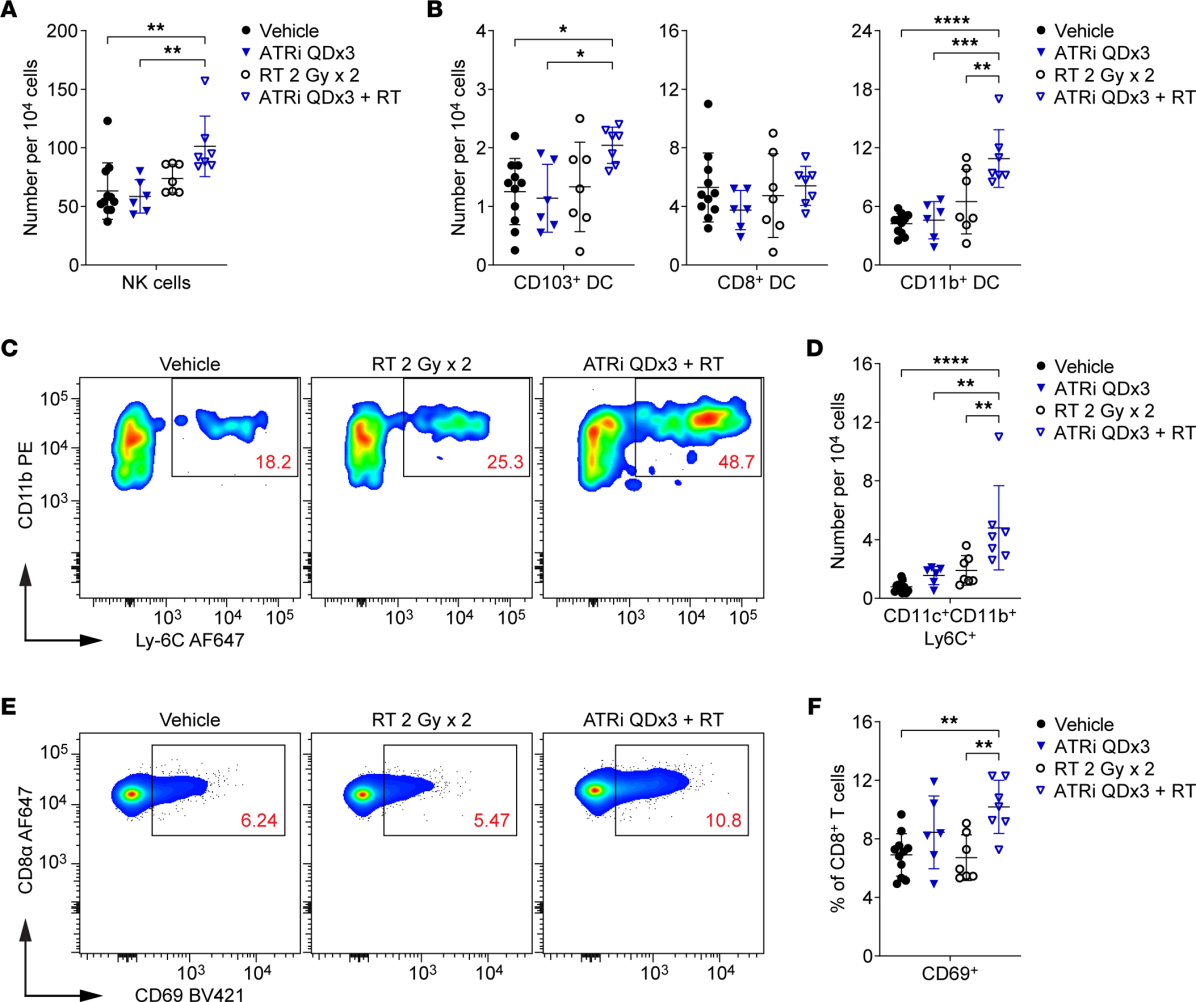
Short-course ATRi plus RT promotes accumulation of inflammation-associated innate immune cells and increased CD8^+^ T cell activation in the DLN. (**A**–**F**) CT26 tumor–bearing mice were treated with ATRi QDx3, RT 2 Gy x 2, ATRi QDx3 + RT, or vehicle, and DLNs were immunoprofiled at day 7. (**A**) Quantitation of NK cells (NKp46^+^ and CD3^–^CD19^–^) per 10^4^ cells stained. (**B**) Quantitation of DC subsets per 10^4^ cells stained. Immunoprofiled DC subsets include CD103^+^ DCs (CD11c^+^CD103^+^ and CD3^–^CD19^–^NKp46^–^), CD8^+^ DCs (CD11c^+^CD8^+^ and CD3^–^CD19^–^NKp46^–^CD11b^–^), and CD11b^+^ DCs (CD11c^+^CD11b^+^ and CD3^–^CD19^–^NKp46^–^CD8^–^). (**C**) Representative cytograms depicting CD11b and Ly-6C expression on CD11c^+^ (and CD3^–^CD19^–^NKp46^–^) cells. (**D**) Quantitation of CD11c^+^CD11b^+^Ly-6C^+^ (and CD3^–^CD19^–^NKp46^–^CD8^–^) cells per 10^4^ cells stained. (**E**) Representative cytograms depicting CD69 expression on CD8^+^ T cells. (**F**) Quantitation of newly or recently activated CD69^+^CD8^+^ T cells, as a percentage of CD8^+^ T cells. (**A**, **B**, **D**, and **F**) Data from 2 independent experiments with 3–4 Vehicle, RT, and ATRi QDx3 + RT mice per group and 1 experiment with 3 Vehicle and 6 ATRi QDx3 mice per group. *n* = 11 Vehicle, 6 ATRi QDx3, 7 RT, 7 ATRi QDx3 + RT. Mean and SD bars shown. *P < 0.05, **P < 0.01, ***P < 0.001, ****P < 0.0001 by ANOVA with Tukey’s multiple-comparison test.

Next we investigated whether increased proinflammatory signaling in the TME was associated with changes in the DLN at day 7. We immunoprofiled DLN from ATRi QDx3 plus RT–treated and RT-treated mice, and a subset of the vehicle-treated mice, whose tumors were assayed for cytokines/chemokines. We also immunoprofiled DLN from ATRi QDx3–treated and vehicle-treated mice from separate cohorts. We quantified NK cells (NKp46^+^) and total DCs (CD11c^+^ and NKp46^–^) within the non–T cell and non–B cell (CD3^–^CD19^–^) populations. ATRi QDx3 plus RT increased the relative number of NK cells in the DLN compared with ATRi QDx3 alone and vehicle control, but did not alter the total DC or CD3^–^CD19^–^ cell populations (Figure 5A and Supplemental Figure 5D).

ATRi QDx3 plus RT did not alter the relative number of CD8^+^ DCs (CD11c^+^CD8^+^CD11b^–^), but did increase CD103^+^ DCs relative to ATRi QDx3 alone and vehicle control (Figure 5B). ATRi QDx3 plus RT strikingly increased the relative number of CD11b^+^ DCs (CD11c^+^CD11b^+^CD8^–^) compared with all other treatment groups (Figure 5B). We further examined the CD11c^+^CD11b^+^ population for expression of Ly-6C (Figure 5C). CD11c^+^CD11b^+^Ly-6C^+^ cells are monocyte-lineage cells that arise at sites of inflammation and that fall along the differentiation spectrum between inflammatory monocytes and monocyte-derived DCs with functional antigen-presenting ability (42–46). ATRi QDx3 plus RT increased the relative number of CD11c^+^CD11b^+^Ly-6C^+^ cells in the DLN at day 7 compared with all other treatment groups (Figure 5D). These data suggest that ATRi QDx3 plus RT promotes migration of inflammation-associated innate immune cells to the DLN.

We also immunoprofiled CD8^+^ T cells to examine T cell priming and activation in the DLN at day 7, using expression of the early activation marker CD69 to identify newly or recently activated CD8^+^ T cells (47) (Figure 5E). ATRi QDx3 plus RT increased the percentage of the CD69^+^CD8^+^ T cells in the DLN at day 7 compared with vehicle and RT alone (Figure 5F). ATRi QDx3 treatment resulted in a mean increase in CD69^+^CD8^+^ T cells compared with vehicle control that was not statistically significant (Figure 5F), but that coincided with the timing of the proliferative rebound observed after ATRi cessation. While this suggests that CD8^+^ T cell recovery after ATRi cessation does contribute to the increase in CD69^+^CD8^+^ T cells at day 7, our data collectively show that ATRi QDx3 plus RT promotes priming and activation of CD8^+^ T cells, concomitant with increased accumulation of inflammation-associated innate immune cells, in the DLN.

### Prolonged daily ATRi potentiates RT-induced IFN-β but not inflammatory chemokines in the tumor microenvironment and promotes CD8^+^ T cell activation in the DLN.

**Figure 6:**
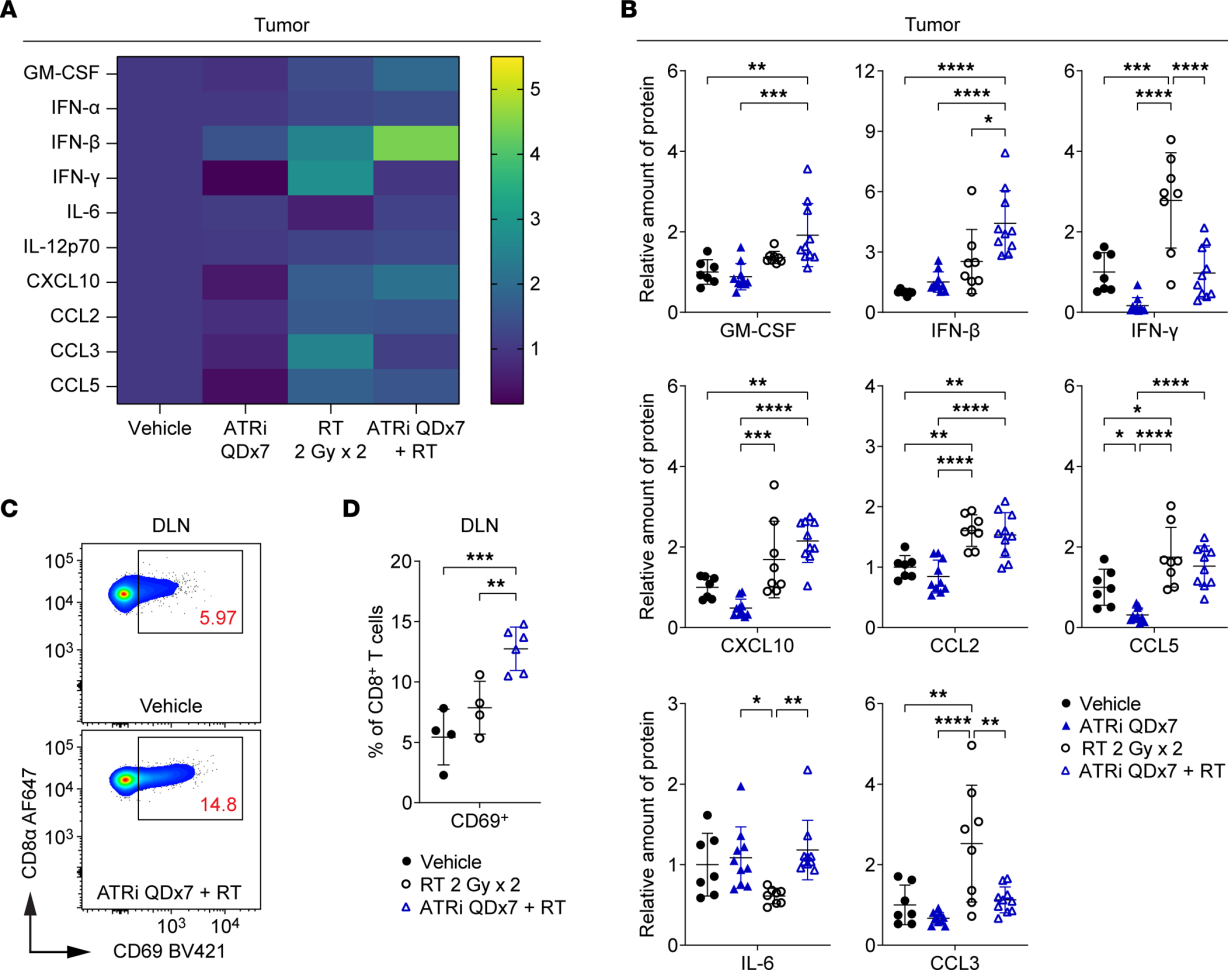
Prolonged daily ATRi potentiates RT-induced IFN-β but not inflammatory chemokines in the tumor microenvironment. (**A**–**D**) CT26 tumor–bearing mice were treated with ATRi on days 1–7 (ATRi QDx7), RT 2 Gy x 2 (days 1–2), ATRi QDx7 + RT, or vehicle. (**A**) Heatmaps depicting the mean relative amount (normalized to vehicle control) of 10 inflammatory cytokines and chemokines in tumors at day 7. (**B**) Quantitation of the relative amount of protein (normalized to vehicle control) for a subset of inflammatory cytokines and chemokines in tumors at day 7. (**A** and **B**) Data from at least 4 independent experiments with 1–4 mice per group. *n* = 7 Vehicle, 10 ATRi QDx7, 8 RT, 10 ATRi QDx7 + RT. (**C** and **D**) CT26 tumor–bearing mice were treated with RT 2 Gy x 2, ATRi QDx7 + RT, or vehicle. (**C**) Representative cytograms depicting CD69 expression on CD8^+^ T cells in DLN at day 7. (**D**) Quantitation of newly or recently activated CD69^+^CD8^+^ T cells in the DLN at day 7. Data from at least 2 independent experiments with 1–3 mice per group. *n* = 4 Vehicle, 4 RT, 6 ATRi QDx7 + RT. (**B** and **D**) Mean and SD bars shown. *P < 0.05, **P < 0.01, ***P < 0.001, ****P < 0.0001 by ANOVA with Tukey’s multiple-comparison test.

Our data demonstrate that cessation of short-course, 3-day ATRi treatment triggers a proliferative rebound in T cell populations, and, in parallel, ATRi QDx3 integrates with RT to increase inflammatory cytokine/chemokine signaling in the TME. Next, we sought to determine how prolonged, daily ATRi treatment for 7 consecutive days (ATRi QDx7) impacts RT-induced proinflammatory signaling in the TME at day 7 (Figure 6A). Similarly to ATRi QDx3 plus RT, ATRi QDx7 plus RT increased IFN-β in the TME compared with all other treatments, attenuated RT-induced IFN-γ, preserved IL-6 levels at baseline, and did not alter IFN-α (Figure 6B and Supplemental Figure 6). In contrast to short-course ATRi treatment, ATRi QDx7 plus RT did not potentiate RT-induced CXCL10 and CCL2 (Figure 6B). In addition, ATRi QDx7 alone reduced CCL5 compared with all other treatments, and ATRi QDx7 attenuated RT-induced CCL3 expression (Figure 6B).

### Prolonged daily ATRi abolishes the adaptive T cell response and expansion of tumor antigen–specific CD8^+^ T cells in the periphery following RT.

**Figure 7:**
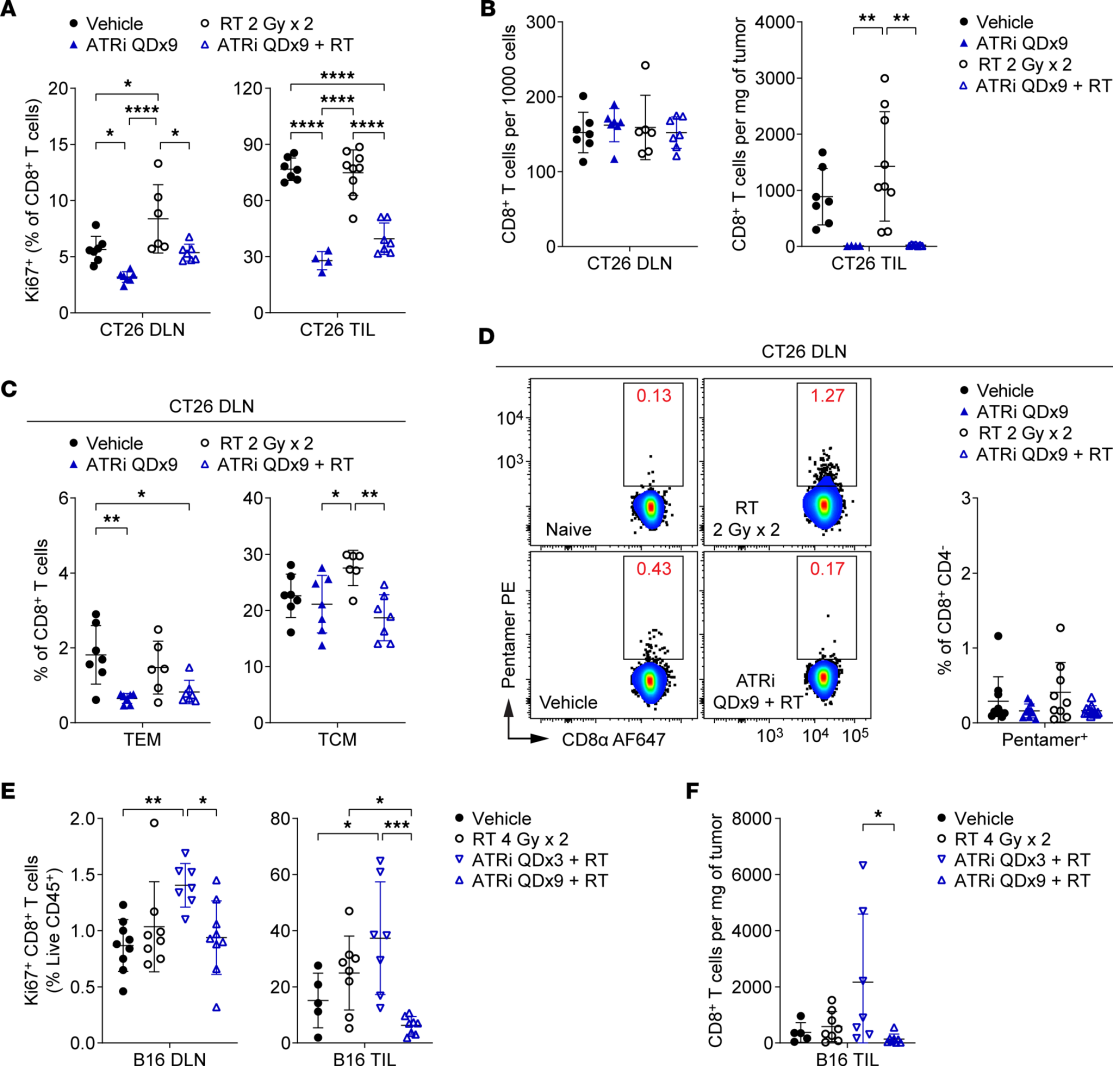
Prolonged daily ATRi treatment abolishes the adaptive CD8^+^ T cell response following RT. (**A**–**D**) CT26 tumor–bearing mice were treated with ATRi on days 1–9 (ATRi QDx9), RT 2 Gy x 2 (days 1–2), ATRi QDx9 + RT, or vehicle, and immunoprofiled at day 9, 1 hour after the final dose of ATRi. (**A**) Quantitation of proliferating (Ki67^+^) CD8^+^ T cells, as percentages of CD8^+^ T cells, in DLN and TILs. (**B**) Quantitation of CD8^+^ T cells in DLN (per 1,000 cells) and TILs (per milligram of tumor). (**C**) Quantitation of CD8^+^ Tem and Tcm cells, as percentages of CD8^+^ T cells, in DLN. (**A**–**C**) Data from 2 independent experiments (1 for ATRi QDx9 TIL) with 2–5 mice per group. *n* = 7 Vehicle, 7 ATRi QDx9 DLN (4 TIL), 9 RT TIL (6 DLN), 7 ATRi QDx9 + RT. (**D**) Representative cytograms and quantitation of Pentamer^+^ CD8^+^ T cells, as percentages of CD8^+^CD4^–^ cells, in DLN. Data from 5 independent experiments with 1–4 mice per group. *n* = 10 Vehicle, 9 ATRi QDx9, 9 RT, 12 ATRi QDx9 + RT. (**E** and **F**) B16 tumor–bearing mice were treated with RT 4 Gy x 2 (days 1–2), ATRi QDx3 + RT, ATRi QDx9 + RT, or vehicle, and immunoprofiled at day 9 (1 hour after the final dose of ATRi QDx9). (**E**) Quantitation of proliferating (Ki67^+^) CD8^+^ T cells, as percentages of live, CD45^+^ immune cells, in DLN and TILs. (**F**) Quantitation of CD8^+^ T cells, per milligram of tumor, in TILs. (**E** and **F**) Data from 2 independent experiments with 2–6 mice per group. *n* = 9 Vehicle DLN (5 TIL), 8 RT, 7 ATRi QDx3 + RT, 9 ATRi QDx9 + RT DLN (8 TIL). (**A**–**F**) Mean and SD bars shown. *P < 0.05, **P < 0.01, ***P < 0.001, ****P < 0.0001 by ANOVA with Tukey’s multiple-comparison test.

Next we investigated the consequences of prolonged, daily ATRi on proliferating T cell populations in the DLN and TILs. We treated mice with ATRi for 9 consecutive days (ATRi QDx9), with or without RT (2 Gy x 2), and immunoprofiled DLN and TILs at day 9, 1 hour after the last dose of ATRi. ATRi QDx9 treatment, alone or with RT, reduced proliferating CD8^+^ T cells in both the DLN and TILs (Figure 7A). ATRi QDx9 had similar impacts on proliferating CD4^+^ Tconvs and Tregs (Supplemental Figure 7A). The relative numbers of CD8^+^ T cells or CD4^+^ Tconvs in the DLN were not changed with ATRi QDx9 or ATRi QDx9 plus RT treatment (Figure 7B and Supplemental Figure 7B). However, ATRi QDx9, independent of RT, reduced relative Treg numbers in the DLN (Supplemental Figure 7B) and reduced overall spleen weight (Supplemental Figure 7C), providing further support that the impact of ATRi treatment may differ among T cell and other immune cell populations. Strikingly, ATRi QDx9 rendered TILs essentially devoid of CD8^+^ T cells and Tregs following treatment (Figure 7B and Supplemental Figure 7B). Similarly, the TILs of ATRi QDx9–treated and ATRi QDx9 plus RT–treated mice had fewer CD4^+^ Tconvs compared with vehicle-treated and RT-treated mice (Supplemental Figure 7B). Therefore, prolonged, 9-day ATRi treatment results in a dearth of proliferating and total T cells in the TILs at day 9.

Given the persistent suppression of proliferating T cell populations, we anticipated that ATRi QDx9 would abolish CD8^+^ Tem and tumor antigen–specific CD8^+^ T cell expansions in the DLN at 1 week after RT. In contrast to ATRi QDx3, ATRi QDx9, independent of RT, resulted in reduced CD8^+^ Tem cells in DLN compared with vehicle and reduced CD8^+^ Tcm cells compared with RT alone (Figure 7C). Accordingly, the CD8^+^ Tn cell pools in the DLN were increased by ATRi QDx9, alone or with RT, compared with RT alone (Figure 7D). Next we examined the DLN for tumor antigen–specific CD8^+^ T cells at day 9 (Figure 7D). We observed no significant differences across treatments in the numbers of total CD8^+^CD4^–^ cells (Supplemental Figure 7E) or in the percentages of Pentamer^+^ CD8^+^ T cells in the DLN (Figure 7D). Therefore, prolonged daily ATRi treatment for 9 consecutive days reduces activated CD8^+^ T cell populations (Tem and Tcm) and abolishes the expansion of both CD8^+^ Tem and tumor antigen–specific CD8^+^ T cells in the DLN at day 9, one week after RT.

To confirm that limitation of the post-therapy CD8^+^ T cell response by ATRi QDx9 is not restricted to the CT26 tumor model or to the BALB/c mouse strain, we examined proliferating (Ki67^+^) CD8^+^ T cells in the DLN and TILs of B16 tumor–bearing C57BL/6 mice treated with two 4 Gy fractions of tumor-targeted radiation (RT 4 Gy x 2), ATRi QDx3 plus RT, ATRi QDx9 plus RT, or vehicle. Compared with vehicle control, ATRi QDx3 plus RT increased proliferating CD8^+^ T cells (as a percentage of live, CD45^+^ immune cells) in the DLN and TILs of B16 tumor–bearing mice, while ATRi QDx9 plus RT did not (Figure 7E). Furthermore, treatment with ATRi QDx3 plus RT resulted in more CD8^+^ T cells in the tumor infiltrate than treatment with ATRi QDx9 plus RT, which, like in the CT26 model, nearly depleted the tumor of CD8^+^ T cells (Figure 7F). Therefore, ATRi schedule considerations extend beyond a single tumor model.

### Cessation of ATRi treatment is necessary for extended survival after ATRi combined with sequential RT plus PD-L1 blockade.

Next we compared the efficacy of short-course versus prolonged ATRi in combination with sequential RT plus anti–PD-L1 in the CT26 model. Sequential scheduling of RT and PD-L1 was chosen for two reasons. First, CT26 is highly responsive to concurrent treatment with RT and anti–PD-L1, but is less responsive to sequential therapy with anti–PD-L1 started 1 week after fractionated RT (48), enabling us to better define the impact of ATRi on efficacy of RT plus anti–PD-L1. Second, ATRi QDx3 plus RT caused increased CD8^+^ T cell proliferation and expansion of tumor antigen–specific CD8^+^ T cells in the DLN 5–7 days after RT.

**Figure 8:**
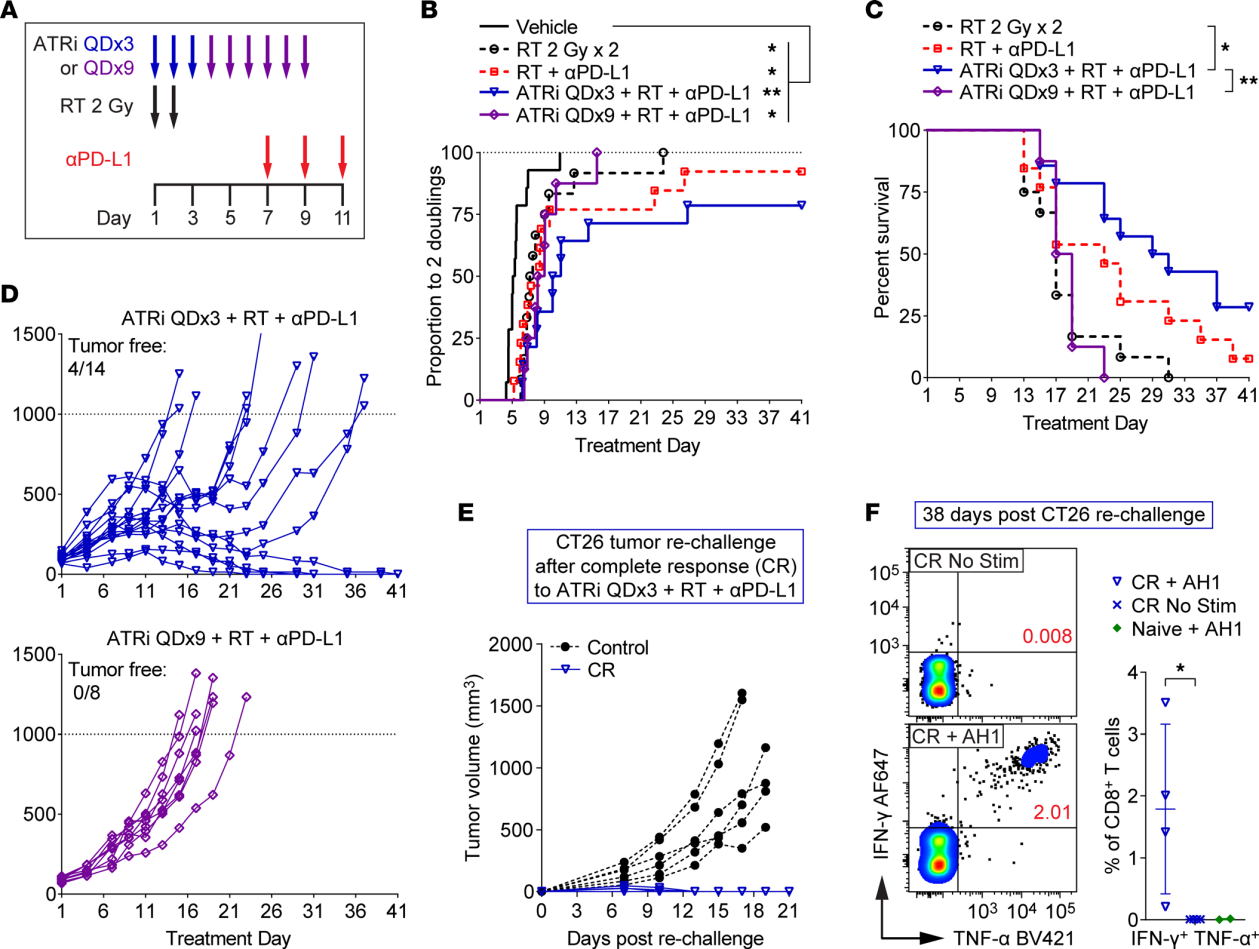
ATRi cessation is necessary for extended survival and complete responses after treatment with ATRi and sequential RT plus PD-L1 blockade. (**A**) Schema for CT26 tumor–bearing mice treated with RT 2 Gy x 2 (days 1–2), RT + anti–PD-L1 (αPD-L1; days 7, 9, 11), ATRi QDx3 + RT + αPD-L1, ATRi QDx9 + RT + αPD-L1, or vehicle. (**B**) Proportions of mice that reached 2 tumor volume doublings, plotted over time. *P < 0.05, **P < 0.01 by log-rank test with Holm-Šidák adjustment for multiple comparisons (all groups). (**C**) Survival curves for non-vehicle groups. The survival endpoint was when tumor volume exceeded 1,000 mm^3^. *P < 0.05, **P < 0.01 by log-rank test with Holm-Šidák adjustment for multiple comparisons (non-vehicle groups). (**D**) Individual tumor growth curves for the ATRi QDx3 + RT + αPD-L1 and ATRi QDx9 + RT + αPD-L1 groups. The numbers of tumor-free mice (complete responders) are noted. (**A**–**D**) Data from 2 independent experiments with 5–8 mice per group (1 experiment for ATRi QDx9 + RT + αPD-L1). *n* = 14 Vehicle, 12 RT, 13 RT + αPD-L1, 14 ATRi QDx3 + RT + αPD-L1, and 8 ATRi QDx9 + RT + αPD-L1. (**E**) Individual tumor growth curves for ATRi QDx3 + RT + αPD-L1 complete responders (CR, *n* = 4) rechallenged with CT26 and for control mice (*n* = 6) challenged with CT26 for the first time. (**F**) Representative cytograms and quantitation of IFN-γ^+^TNF-α^+^ CD8^+^ T cells in AH1 peptide–stimulated (CR + AH1, *n* = 4) versus unstimulated (CR No Stim, *n* = 4) splenocytes from CR mice and AH1 peptide–stimulated splenocytes from tumor-naive mice (Naive + AH1, *n* = 2). *P > 0.05 by 2-tailed, unpaired *t* test. Naive + AH1 controls were not included in the statistical analysis.

Mice received RT (2 Gy x 2), RT plus anti–PD-L1 (10 mg/kg, days 7, 9, and 11), ATRi QDx3 plus RT plus anti–PD-L1, ATRi QDx9 plus RT, or vehicle (Figure 8A). We observed considerable variation in tumor responses among mice within treatment groups, particularly in the RT plus anti–PD-L1 and ATRi QDx3 plus RT plus anti–PD-L1 treatment arms, with a number of mice in each arm reaching the survival endpoint (tumor volume >1,000 mm^3^) much sooner than other mice within that group (Supplemental Figure 8). Therefore, we determined the time (days) to 2 tumor volume doublings for each animal and compared the proportion of mice reaching 2 tumor doublings across treatment groups. All treatments delayed the time to 2 tumor doublings when compared with vehicle control (Figure 8B). Since 9 of 14 vehicle mice reached the survival endpoint by day 13, no mice in this arm were continued past day 13. Loss of animals within treatment groups at later time points (at which other mice in the same group exhibited responses) impaired our ability to use tumor growth curves to compare treatment efficacy. Therefore, we examined survival differences among the treatment groups.

Sequential RT plus anti–PD-L1 extended median survival from 17 days to 23 days versus RT alone, but the survival difference did not reach statistical significance (Figure 8C). Addition of ATRi QDx3 to RT plus anti–PD-L1 extended median survival to 30 days, significantly longer than with RT alone (Figure 8C). While the difference in survival following ATRi QDx3 plus RT plus anti–PD-L1 versus RT plus PD-L1 did not reach statistical significance, the addition of ATRi QDx3 increased the number of complete responses (complete tumor clearance) from 1 of 13 mice treated with RT plus anti–PD-L1 to 4 of 14 mice treated with ATRi QDx3 plus RT plus anti–PD-L1 (Figure 8D and Supplemental Figure 8).

Importantly, the survival benefit of ATRi plus RT plus anti–PD-L1 was abolished when ATRi treatment was continued for 9 days rather than ceased after 3 days. Median survival after ATRi QDx9 plus RT plus anti–PD-L1 was 18 days, similar to median survival after RT alone and significantly shorter than the 30-day median survival after ATRi QDx3 plus RT plus anti–PD-L1 (Figure 8C). In addition, ATRi QDx9 plus RT plus anti–PD-L1 did not yield any tumor-free mice (Figure 8D). Therefore, cessation of ATRi is necessary to extend survival and promote complete responses after ATRi plus RT plus anti–PD-L1, as prolonged, daily ATRi treatment antagonizes treatment efficacy.

To confirm that mice exhibiting complete responses to ATRi QDx3 plus RT plus anti–PD-L1 have immunologic memory against CT26, complete responder (CR) mice were rechallenged with CT26 cells in the contralateral flank after a tumor-free period of greater than 100 days. CR mice rapidly rejected CT26 tumor growth, while tumors grew in control mice challenged with CT26 for the first time (Figure 8E). To confirm that this immunologic memory involved CD8^+^ T cells that recognize CT26 tumor antigen, splenocytes were harvested from CR mice 38 days after initiation of the rechallenge and were stimulated ex vivo with AH1 peptide for 24 hours. AH1 peptide exposure stimulated a pool of CD8^+^ T cells from CR mice (CR + AH1) to produce IFN-γ and TNF-α (Figure 8F). Importantly, IFN-γ^+^TNF-α^+^ CD8^+^ T cells were not present in unstimulated splenocytes from CR mice (CR No Stim) or in stimulated splenocytes from tumor-naive control mice (Naive + AH1) (Figure 8F), indicating the presence of a tumor antigen–specific CD8^+^ T cell memory pool in spleens of CR mice.

## Discussion

Established tumors contain multiple subpopulations of T cells in various degrees of function or dysfunction, including effector T cells, exhausted T cells, and regulatory T cells (49–52). The proliferation of these multiple subpopulations of T cells may be an unappreciated target of ATRi. Consistent with this premise, we show that ATRi reduces the existing proliferating T cell pool in both the tumor and DLN and that, with the appropriate sequence and combination treatment, this generates a niche in which new, tumor-reactive CD8^+^ T cells can expand. We also show that cessation of ATRi results in a rapid, but nonselective, proliferative rebound in T cell populations within 4 days. When short-course ATRi is integrated with RT, treatment elicits preferential expansion of tumor antigen–specific CD8^+^ T cells in the DLN, the lymphoid site in tumor-bearing mice that is crucial for the efficacy of PD-1/PD-L1 checkpoint blockade (32). A significant portion of the expanding tumor antigen–specific CD8^+^ T cells in the DLN are effector/effector memory (Tem) cells, which may infiltrate tumors and drive response to immune checkpoint blockade (53).

Our findings that prolonged daily ATRi abolishes expansion of tumor-specific CD8^+^ T cells in the DLN and abolishes the survival benefits afforded by addition of short-course ATRi and sequential anti–PD-L1 treatment to RT show that ATRi must be ceased to allow a CD8^+^ T cell–dependent adaptive immune response. Our prior work in a genetically engineered mouse model of lung adenocarcinoma showed that antitumor responses to ATRi plus RT manifest over several weeks after ATRi treatment ceased (17). While the overall duration of ATRi and RT treatment was longer than in our current studies, ATRi treatment was ceased at the end of the RT regimen, and this was associated with a CD8^+^ T cell–dependent antitumor response. These observations are impactful as active clinical trials of immunotherapy combined with ATRi treat with ATRi twice daily for up to 2 weeks, with cycles repeated monthly (ClinicalTrials.gov) (54). Our data suggest that ATRi cessation is critical to allow for CD8^+^ T cell responses to immune checkpoint blockade, and that shorter cycles of ATRi should be investigated.

Our data show that the expansion of tumor antigen–specific CD8^+^ T cells following short-course ATRi plus RT is driven by proinflammatory cytokine and chemokine signaling in the TME and downstream communication of that signaling (via immune cell migration) to the DLN. Both ATRi schedules potentiated RT-induced IFN-β signaling in the TME. However, only short-course ATRi potentiates RT-induced increases of the IFN-β–inducible, proinflammatory chemokines CXCL10 and CCL2. Therefore, the duration of ATRi treatment that is integrated with RT affects the post-RT chemokine milieu in the TME. The dependence of both innate and adaptive immune responses after RT on IFN-β signaling in the TME is well established (55, 56), and increases of IFN-β, CXCL10, and CCL2 in tumors affect immune cell recruitment and activation. In the short term, IFN-β signaling positively regulates NK cell function, DC maturation, and T cell activation and modulates differentiation of Ly-6C^+^ inflammatory monocytes (57, 58). IFN-β also induces production of CXCL10, which recruits antitumor NK cells and effector T cells, and CCL2, which triggers the initial recruitment of inflammatory monocytes (57–60). Prior reports have shown that IFN-β, CXCL10, and CCL2 are increased after ATRi plus RT in the TME (19) and in tumor cells cultured in vitro (26), and this has been attributed to tumor cell–intrinsic signaling. However, in response to proinflammatory stimuli, CXCL10 can be secreted by T cells and monocytes and CCL2 can be secreted by myeloid lineage cells including monocytes, macrophages, and DCs (57, 59, 61). Therefore, it is possible that tumor-infiltrating myeloid cells are an important source of CXCL10 and CCL2 after short-course ATRi plus RT, and that prolonged daily ATRi negatively impacts these myeloid cells and reduces CXCL10 and CCL2 production.

Consistent with increased tumor inflammation, short-course ATRi plus RT increases migration of inflammation-associated cells to the DLN. A recent report identified an important role for NK cells in antitumor immune responses following treatment with ATRi, RT, and immune checkpoint blockade (62), and we observe that NK cells accumulate in the DLN after short-course ATRi plus RT. In addition, we show that short-course ATRi plus RT causes CD103^+^ DCs and CD11c^+^CD11b^+^Ly-6C^+^ cells to accumulate in the DLN at day 7. CD103^+^ DCs are key mediators of antitumor immune responses that traffic tumor antigens to the DLN and cross-present antigen to CD8^+^ T cells (63–65). Ly-6C–expressing CD11c^+^CD11b^+^ cells arise at inflamed tissues and reside along a differentiation spectrum between inflammatory monocytes and antigen-presenting, monocyte-derived DCs (moDCs) (42–46). Inflammatory monocytes can phagocytose antigens, migrate to the lymph node, and differentiate into antigen-presenting moDCs, which then present antigen to CD4^+^ T cells, cross-present antigen to CD8^+^ T cells, or transfer antigen to lymph node–resident DCs, which, in turn, cross-present to CD8^+^ T cells (42–44, 66, 67). Preclinical evidence supports that moDCs in the tumor and DLN play a key role in CD8^+^ T cell activation and antitumor immunity after immunotherapy (46), and IFN-β signaling in tumor-infiltrating inflammatory monocytes has been associated with T cell expansion in patients who respond to immune checkpoint blockade (68). We cannot definitively state that the CD11c^+^CD11b^+^Ly-6C^+^ cells we identified in the DLN are antigen-presenting moDCs that prime CD8^+^ T cells. However, our data associate accumulation of these cells in the DLN after short-course ATRi plus RT with increased CD8^+^ T cell activation at day 7 and subsequent tumor antigen–specific CD8^+^ T cell expansion at day 9.

To summarize, our work herein highlights the importance of proper scheduling of ATRi for immune modulation. We associate the expansion of tumor antigen–specific CD8^+^ T cells after short-course ATRi plus RT (Figure 1) with both the potentiation of RT-induced tumor inflammation (Figure 4) and innate immune activity (Figure 5) by ATRi as well as the independent effects of ATRi on T cells and the proliferative rebound that follows cessation of ATRi (Figures 2 and 3). Despite potentiating RT-induced IFN-β in tumors (Figure 6), prolonged daily ATRi treatment abolishes the peripheral CD8^+^ T cell response after RT and depletes the tumor infiltrate of CD8^+^ T cells, in both the CT26 and B16 models (Figure 7). Moreover, prolonged daily ATRi abolishes the peripheral expansion of tumor antigen–specific effector CD8^+^ T cells (Figure 7) that otherwise occurs after short-course ATRi treatment integrated with RT. Moreover, prolonged daily ATRi treatment negates the therapeutic benefit of adding short-course ATRi to sequential anti–PD-L1 after RT (Figure 8). ATRi cessation may be important for innate immune responses as well, as only short-course ATRi potentiated RT-induced CXCL10 and CCL2 (Figure 4).

Therefore, in 2 syngeneic tumor models, ATRi cessation is essential to allow for T cell recovery and for increases in proliferating CD8^+^ T cells after ATRi plus RT. In CT26, ATRi cessation is essential for the expansion of tumor-specific CD8^+^ T cell clones after RT. CT26 is a well-characterized and robust preclinical model in which to study immune-modulating therapies. The oligoclonal nature of CD8^+^ T cell responses against the CT26 tumor antigen AH1 in mice treated with immunotherapy (34) makes the CT26 model an excellent system in which to study the impact of a clinically significant variable, the duration of ATR inhibitor treatment, on tumor antigen–specific CD8^+^ T cell responses. Our data argue that cessation of ATRi is essential to allow CD8^+^ T cell responses to regimens that combine ATRi with RT and/or immune checkpoint inhibitors in the clinic.

## Methods

### Cell lines and reagents.

CT26 (CRL-2638) and B16-F10 (CRL-6475) were from ATCC and cultured in RPMI (Lonza or Gibco) or DMEM (Lonza), respectively, containing 10 % FBS (Gemini Bio) and 100 U/mL penicillin plus 100 mg/mL streptomycin (Lonza). Cells were tested for mycoplasma. AZD6738 (ATRi) was provided by AstraZeneca and dosed at 75 mg/kg by oral gavage as described previously (15, 17). FTY720 (Fingolimod hydrochloride, MilliporeSigma) was dissolved in sterile normal saline (0.9% sodium chloride) and dosed at 1 mg/kg via intraperitoneal injection. InVivoPlus anti–mouse PD-L1 antibody (clone 10F.9G2, Bio X Cell) was diluted to 1 mg/mL in InVivoPure pH 6.5 Dilution Buffer (Bio X Cell) and dosed at 10 mg/kg via intraperitoneal injection. AH1 peptide (SPSYVYHQF, Anaspec) was dissolved in DMSO at 20 mg/mL and diluted to a final concentration of 10 μg/mL in T Cell Media (RPMI, 10 % FBS, 100 U/mL penicillin plus 100 mg/mL streptomycin, and the following [all from Gibco]: 1× MEM–nonessential amino acids, 1 mM sodium pyruvate, 5 mM HEPES pH 8.0, 50 mM β-mercaptoethanol).

### Mice and treatments.

Female BALB/c and female C57BL/6 mice (6–8 weeks old) were from The Jackson Laboratory. CT26 (~5 × 10^5^ cells) in RPMI or B16-F10 (2.5 × 10^5^ cells) in DMEM was subcutaneously injected into the right hind flank of 8- to 10-week-old mice. Treatment was initiated when tumors reached about 60–120 mm^3^ (CT26) or about 55–135 mm^3^ (B16-F10). For CT26 tumor irradiation, immobilized mice received 2 fractions of 2 Gy (6 mV photon energy, 2 cm field) on days 1–2. For B16 tumor irradiation, isoflurane-anesthetized mice received 2 fractions of 4 Gy using an image-guided Precision SmART+ (225 kV) with 10 mm collimator and Cu treatment filter. Mice received 75 mg/kg AZD6738 45–60 minutes before irradiation on days 1–2 and a third dose, approximately 18 hours later (CT26) or 24 hours later (B16), on day 3. Subsequent doses of AZD6738 for prolonged daily treatment experiments were every 24 hours thereafter. For FTY720 Q2Dx4 experiments, FTY720 was administered alongside AZ6738 before radiation on day 1, and again on days 3, 5, and 7. For anti–PD-L1 tumor response experiments, anti–PD-L1 was administered on days 7, 9, and 11. Tumors were measured at least twice weekly with calipers, and volumes calculated as volume = (length × width^2^)/2. The survival endpoint was designated as tumor volume >1,000 mm^3^.

### Tissue processing, staining, and multiparameter flow cytometry for immunoprofiling.

CT26 and B16 tumors and tumor-draining (right inguinal) lymph nodes (DLNs) were harvested from mice at day 4, 7, or 9. For experiments involving tumor (TIL) immunoprofiling (except CT26 FTY720 and B16 experiments), splenocytes were used for single-color controls, fluorescence-minus-one (FMO) controls, and general gating. Tumors were weighed before processing. Single-cell suspensions were generated from tissues as follows: DLNs and spleens were mechanically dissociated between frosted glass slides and filtered through 70 μm cell strainers (Corning). CT26 tumor tissue (≤300 mg) was injected in multiple sites with 1.5 mL Liberase digestion solution (50 μg/mL Liberase DL research grade [Roche] and 10 U/mL DNase I [MilliporeSigma] in RPMI), incubated 3 minutes at room temperature, cut into small pieces, incubated in a total volume of 5 mL Liberase digestion solution for 15 minutes at 37°C, mechanically dissociated between frosted glass slides, filtered through 70 μm cell strainers, vortexed at low speed for 90 seconds, and filtered again through new 70 μm cell strainers. B16 tumor tissue (≤200 mg) was minced, digested in 2 mL 1× Dri Tumor & Tissue Dissociation Reagent (BD Biosciences) and quenched according to the manufacturer’s protocol, and filtered through 70 μm cell strainers. Erythrocytes were lysed in 150 mM NH_4_Cl, 10 mM NaHCO_3_, 0.1 mM EDTA pH 8.0 for 10 seconds (tumors) or 30 seconds (spleens).

Cell suspensions were counted using a Scepter 2.0 or 3.0 handheld counter (Millipore) or Cellometer K2 (Nexcelom) and seeded at 1.5 × 10^6^ to 2 × 10^6^ cells (equivalent density within a given experiment) in 96-well round-bottom plates for staining. Before staining, Fc receptors were blocked for 10 minutes at 4°C with 0.5 μg anti-CD16/32 antibody (TruStain FcX Plus, BioLegend) in FSC buffer (2% FBS/1× PBS). CT26 tumor antigen–specific CD8^+^ T cells were labeled with 10 μL PE-labeled H-2Ld SPSYVYHQF (AH1 peptide) Pro5 MHC Class I Pentamer (ProImmune) in FSC buffer (60 μL total volume) for 10 minutes at room temperature. Lymph nodes from naive (no tumor) negative control mice were included with each experiment, and for AZD6738 QDx9 experiments, PE-labeled HLA-A*02:01 Negative Control Pro5 MHC Class I Pentamer (ProImmune) was included. Cells were then stained with antibodies against surface antigens (in FSC buffer) for 15 minutes at 4°C. Brilliant Stain Buffer Plus (BD Biosciences) was added to antibody cocktails containing multiple Brilliant Violet dye conjugates to prevent polymer dye-dye interactions. True-Stain Monocyte Blocker (BioLegend) was added to antibody cocktails containing PE-Cy7 and/or PerCP-Cy5.5 conjugates to prevent nonspecific binding of monocytes/macrophages to the tandem dyes. After surface staining, dead/dying cells were stained with eFluor 780 viability dye (1:4,000; Thermo Fisher Scientific) in 1× PBS for 10 minutes at 4°C. Samples were then fixed and permeabilized in eBioscience Fixation/Permeabilization reagent (Thermo Fisher Scientific) for 15 minutes at room temperature and, for nuclear (Ki67, Foxp3) or intracellular cytokine (IFN-γ, TNF-α) staining, stained for 45 minutes at room temperature in eBioscience 1× Permeabilization Buffer (Thermo Fisher Scientific) containing antibodies against nuclear/intracellular proteins. For measurement of IFN-γ– and TNF-α–producing CD8^+^ T cells in complete responder or naive (negative) control mice, before staining, splenocytes were stimulated for 24 hours with 10 μg/mL AH1 peptide in T Cell Media, with Golgi-plug (1:1,000; BD Biosciences) added for the final 5 hours. Unstimulated controls for each mouse were also stained. Since T cell receptor ligation induces its internalization, staining for CD3 was performed during intracellular cytokine staining.

For all flow cytometry experiments, uncompensated data were collected using a BD Biosciences LSRFortessa 4-laser cytometer and BD Biosciences FACSDiva software. Compensation and data analyses were performed with FlowJo v10 software (FloJo LLC). Single stained spleen or DLN samples with matching unstained cells or single stained OneComp eBeads (Thermo Fisher Scientific) were used for single-color compensation controls. Fluorescence-minus-one (FMO) controls were used, where appropriate, to empirically determine gating. Reagents for staining panels are included in Supplemental Table 1. Gating strategies are shown in Supplemental Figures 9–14.

### Measurement of intratumoral cytokines and chemokines.

Tumors were harvested at the indicated time points, cut into pieces, and frozen on dry ice. Frozen tumor pieces were weighed, and about 50–60 mg of tumor was added to Precellys CK28-R Protein Safe Hard tissue homogenizing tubes (Bertin Technologies) containing 500 μL per 75 mg tumor of Invitrogen ProcartaPlex Cell Lysis Buffer (Thermo Fisher Scientific) with 1 mM PMSF. A Precellys 24 homogenizer (Bertin Instruments) and the following protocol were used to generate protein extracts: 2 cycles of 6,000 rpm × 15 seconds, samples on ice ≥2 minutes, 2 additional cycles of 6,000 rpm × 15 seconds. Extracts were cleared of insoluble material and frozen in aliquots at –80°C. Protein levels of GM-CSF, IFN-α, IFN-β, IFN-γ, IL-6, IL-12p70, CXCL10 (IP-10), CCL2 (MCP-1), CCL3 (MIP-1α), and CCL5 (RANTES) were measured in tumor extracts using the U-Plex platform (Mesoscale Discovery) according to the manufacturer’s instructions with one modification: after coating the plate, samples were incubated at room temperature with shaking for 1 hour and then at 4°C overnight before antibody detection. GM-CSF, IFN-α, IFN-β, IFN-γ, IL-6, IL-12p70, and CCL5 (RANTES) were assayed in undiluted tumor extract. CXCL10 (IP-10), CCL2 (MCP-1), and CCL3 (MIP-1α) were measured in extracts diluted 1:50 in assay diluent. All samples were assayed in duplicate using a MESO QuickPlex SQ 120 (Mesoscale Discovery). Data analyses and protein concentration determination (in pg/mL) were performed in MSD Discovery Workbench software (Mesoscale Discovery). Protein concentrations were normalized to the mean concentrations of vehicle control samples for a given target, assayed on the same plate, and the data are presented as the relative amount of protein compared with vehicle control. No interplate or inter-run comparisons were made.

### Statistics.

For the complete blood count (CBC) experiment with blood sampling at days 2 and 4, statistical significance was determined by 2-way ANOVA with Šidák’s multiple-comparison test. For FTY720 Q2Dx4 experiments (CBC and immunoprofiling), statistical significance was determined by 1-way ANOVA with Šidák’s multiple-comparison tests comparing vehicle vs. FTY720 Q2Dx4, vehicle vs. ATRi QDx3 plus RT, ATRi QDx3 plus RT vs. ATRi QDx3 plus RT plus FTY720, and FTY720 Q2Dx4 vs. ATRi QDx3 plus RT plus FTY720. For the ATRi QDx3 vs. vehicle cytokine/chemokine experiment, statistical significance was determined by unpaired, 2-tailed *t* test. For CT26 tumor response experiments involving vehicle, RT alone, RT plus anti–PD-L1, ATRi QDx3 plus RT plus anti–PD-L1, and ATRi QDx9 plus RT plus anti–PD-L1 treatment groups, the time (days) at which tumors reached 2 volume doublings was interpolated using Akima spline curves generated from tumor volume data, and significance for comparisons of the proportions of mice reaching 2 tumor volume doublings was determined by log-rank test with Holm-Šidák adjustment for multiple comparisons among all groups. For survival comparisons following treatment with RT alone, RT plus anti–PD-L1, ATRi QDx3 plus RT plus anti–PD-L1, or ATRi QDx9 plus RT plus anti–PD-L1, the survival endpoint was defined as the day when tumor volume exceeded 1,000 mm^3^, and significance was determined by log-rank test with Holm-Šidák adjustment for multiple comparisons among all non-vehicle treatment groups. For all other experiments, statistical significance was determined by ANOVA with Tukey’s multiple-comparison tests. A 95% confidence interval and significance of *P* less than 0.05 were used for all statistical tests. Data are reported as mean ± SD unless otherwise specified. Brackets are shown only for comparisons that were statistically significant unless otherwise specified. All statistical analyses were performed in GraphPad Prism 9.

### Study approval.

Experimental procedures were approved by the University of Pittsburgh Animal Care and Use Committee and performed in accordance with relevant guidelines and regulations.

## Author contributions

FPV, PP, DAC, GMD, JHB, and CJB designed experiments. FPV, SSH, MT, MD, NI, and JC executed experiments. FPV acquired and analyzed data. FPV, DAC, JHB, and CJB wrote the manuscript.

## Supplementary Material

Supplemental data

## Version 1 02/22/2023

Electronic publication
