# Supplementary material for: The schedule of ATR inhibitor AZD6738 can potentiate or abolish antitumor immune responses to radiotherapy
Source: JCI Insight. 2023 Feb 22;8(4):e165615. doi: 10.1172/jci.insight.165615 (PMC9977511; doi:10.1172/jci.insight.165615)
Supplement: Supplemental data [file jciinsight-8-165615-s233.pdf]

SUPPLEMENTAL

Table S1. Antibodies for flow cytometry immunoprofiling

Antibody	Dilution	Manufacturer	Catalog #
Brilliant Stain Buffer Plus	1:5	BD Biosciences	566385
BV650 anti-mouse CD4 (clone GK1.5)	1:500	BD Biosciences	563747
BV786 anti-mouse CD62L (clone MEL-14)	1:500, 1:1000	BD Biosciences	564109
PE-CF594 anti-mouse CD25 (clone PC61)	1:500	BD Biosciences	562695
AF488 anti-mouse CD3 (clone 17A2)	1:250	BioLegend	100212
AF488 anti-mouse CD8α (clone 53-6.7)	1:250	BioLegend	100723
AF488 anti-mouse CD19 (clone 6D5)	1:250	BioLegend	115524
AF488 anti-mouse CD45 (clone 30-F11)	1:250, 1:500	BioLegend	103122
AF488 anti-mouse TCR-β (clone H57-597)	1:250	BioLegend	109215
AF647 anti-mouse IFN-γ (clone XMG1.2)	1:500	BioLegend	505816
AF647 anti-mouse Ki67 (clone 16A8)	1:200	BioLegend	652408
AF647 anti-mouse Ly-6C (clone HK1.4)	1:500	BioLegend	128010
AF647 Rat IgG2a,κ Isotype Control (clone RTK2758)	1:200	BioLegend	400526
AF700 anti-mouse Ki67 (clone 16A8)	1:200	BioLegend	652420
BV421 anti-mouse CD69 (clone H1.2F3)	1:250	BioLegend	104545
BV421 anti-mouse NKp46 (CD335) (clone 29A1.4)	1:50	BioLegend	137611
BV421 anti-mouse TNF-α (clone MP6-XT22)	1:250	BioLegend	506328
BV510 anti-mouse CD4 (clone GK1.5)	1:250, 1:500	BioLegend	100449
BV510 anti-mouse Thy1.2 (CD90.2) (clone 30-H12)	1:250, 1:500	BioLegend	105335
BV785 anti-mouse CD4 (clone GK1.5)	1:500	BioLegend	100453
BV785 anti-mouse CD11c (clone N418)	1:125, 1:250	BioLegend	117336
BV785 anti-mouse CD127 (IL-7Rα) (clone A7R34)	1:100	BioLegend	135037
PE anti-mouse/human CD11b (clone M1/70)	1:250	BioLegend	101207
PE anti-mouse/human KLRG1 (MAFA) (clone 2F1/KLRG1)	1:250	BioLegend	138407
PE-Cy7 anti-mouse CD8α (clone 53-6.7)	1:250, 1:500	BioLegend	100721
PE-Cy7 anti-mouse TCRβ (clone H57-597)	1:250	BioLegend	109221
PerCP-Cy5.5 anti-mouse/human CD44 (clone IM7)	1:500	BioLegend	103031
PerCP-Cy5.5 anti-mouse CD103 (clone 2E7)	1:50	BioLegend	121416
TruStain FcX PLUS anti-mouse CD16/32 (clone S17011E)	1:100	BioLegend	156604
True-Stain Monocyte Blocker	1:20	BioLegend	426103
eFluor450 anti-mouse/rat Foxp3 (clone FJK-16s)	1:200	Invitrogen	48-5773-82
AF647 anti-mouse CD8 (clone KT15)	1:500	MBL Intl	D271-A64
PE-labelled Pro5 MHC I Pentamer (H-2Ld SPSYVYHQF)	10 µL/well	ProImmune	F398-2A/2B
PE-labelled Pro5 MHC I Pentamer (A*02:01 Negative)	10 µL/well	ProImmune	FN01-2A

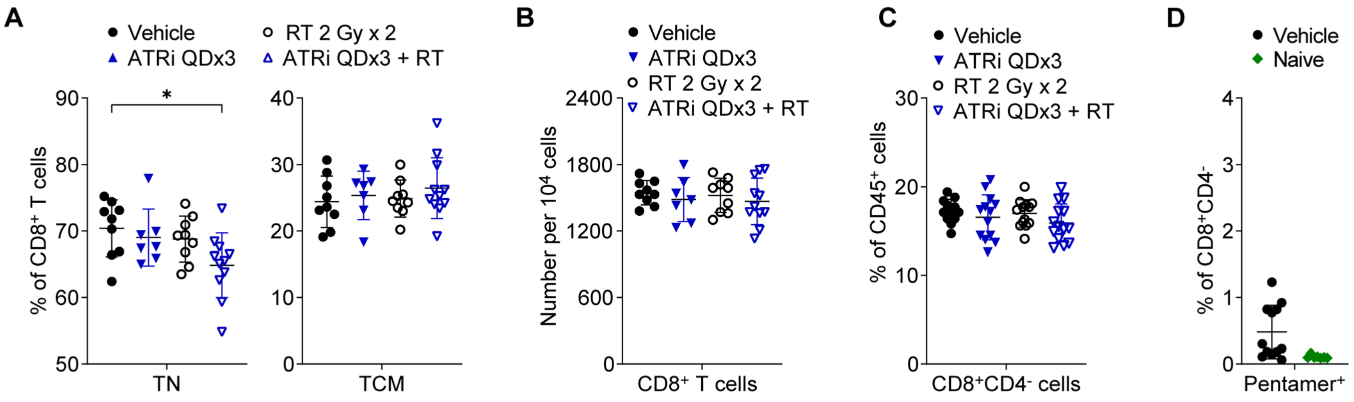

Figure S1. Effects of short-course ATRi integrated with radiotherapy on $CD8^+$ T cells in the tumor-draining lymph node at day 9

CT26 tumor-bearing mice were treated with ATRi on days 1-3 (ATRi QDx3), radiotherapy on days 1-2 (RT 2 Gy x 2), ATRi QDx3 + RT, or vehicle, and tumor-draining lymph nodes (DLN) were immunoprofiled at day 9**. A.** Quantitation of naïve (TN, $CD62L^{hi}CD44^{lo}$) and central memory (TCM, $CD62L^{hi}CD44^{hi}$) $CD8^+$ T cells, as percentages of total $CD8^+$ T cells. **B.** Quantitation of total $CD8^+$ T cells per 10^4 cells stained. **A-B.** Data from at least 4 independent experiments with 1-3 mice per group. n = 9 Vehicle, 7 ATRi QDx3, 9 RT, 11 ATRi QDx3 + RT. **C.** Quantitation of total $CD8^+$ T cells as a percentage of total $CD45^+$ immune cells. **D.** Quantitation of tumor antigen specific $CD8^+$ T cells (Pentamer$^+$), as a percentage of total $CD8^+CD4^-$ cells, in Vehicle-treated mice versus background Pentamer$^+$ in Naïve (no tumor) mice. **C-D**. Data from at least 5 independent experiments with 1-5 mice per group. n = 12 Vehicle, 13 ATRi QDx3, 13 RT, 14 ATRi QDx3 + RT. **A-D**. Mean and SD bars shown. **A-C.** *$p<0.05$ by ANOVA with Tukey's multiple comparisons test.

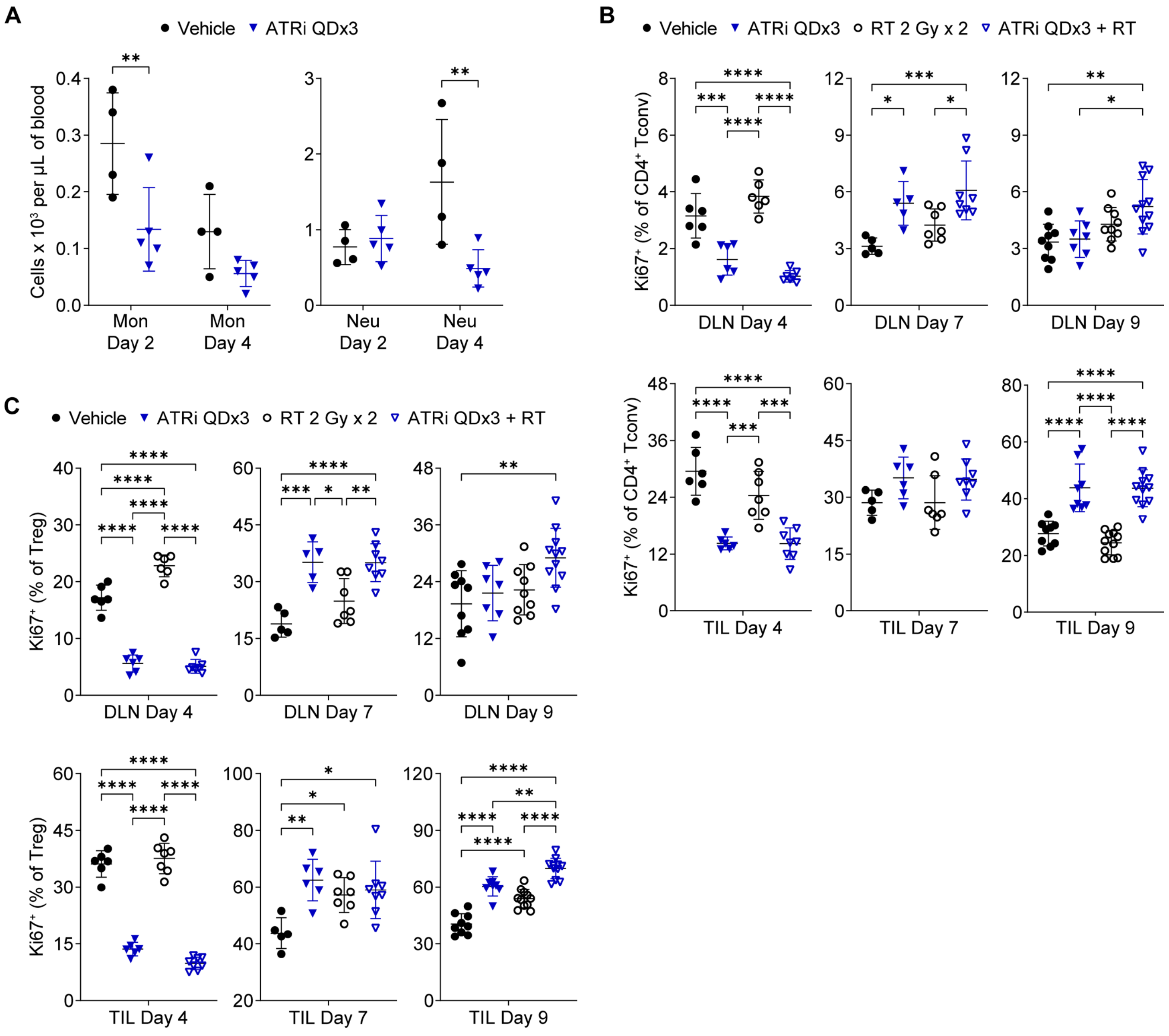

Figure S2. Short-course ATRi impacts peripheral leukocytes and proliferating T cells.
A. BALB/c mice were treated with ATRi QDx3 or vehicle. Complete blood counts (CBC) were performed at Day 2 (24 h after the first dose of ATRi) and Day 4 (24 h after the third dose of ATRi). n = 4 vehicle, 5 ATRi QDx3. Quantitation of total monocytes (Mon) and neutrophils (Neu) per µL of blood are shown. Mean and SD bars shown. **p<0.01 by unpaired, two-way ANOVA with Sidak's multiple comparisons test. **B-C.** CT26 tumor-bearing mice were treated with ATRi QDx3, RT (2 Gy x 2), ATRi QDx3 + RT, or vehicle. DLN and TIL were immunoprofiled at Day 4, Day 7, and Day 9. Proliferating ($Ki67^+$) conventional $CD4^+$ T cells ($CD4^+$ Tconv) (**B**) and Treg (**C**) in the DLN and TIL were quantitated as percentages of the parent population at the depicted time points. Data from at least 3 (Day 4), 2 (Day 7), or 4 (Day 9) independent experiments with 1-4 mice per group. n at Day 4 = 6 Vehicle, 6 ATRi QDx3, 7 RT (6 DLN), 8 ATRi QDx3 + RT (7 DLN). n at Day 7 = 5 Vehicle, 6 ATRi QDx3 (5 DLN), 7 RT, 8 ATRi QDx3 + RT. n at Day 9 = 9 Vehicle, 8 ATRi QDx3 (7 DLN), 11 RT (9 DLN), 11 ATRi QDx3 + RT. Mean and SD bars shown. *p<0.05, **p<0.01, ***p<0.001, ****p<0.0001 by ANOVA with Tukey's multiple comparisons test.

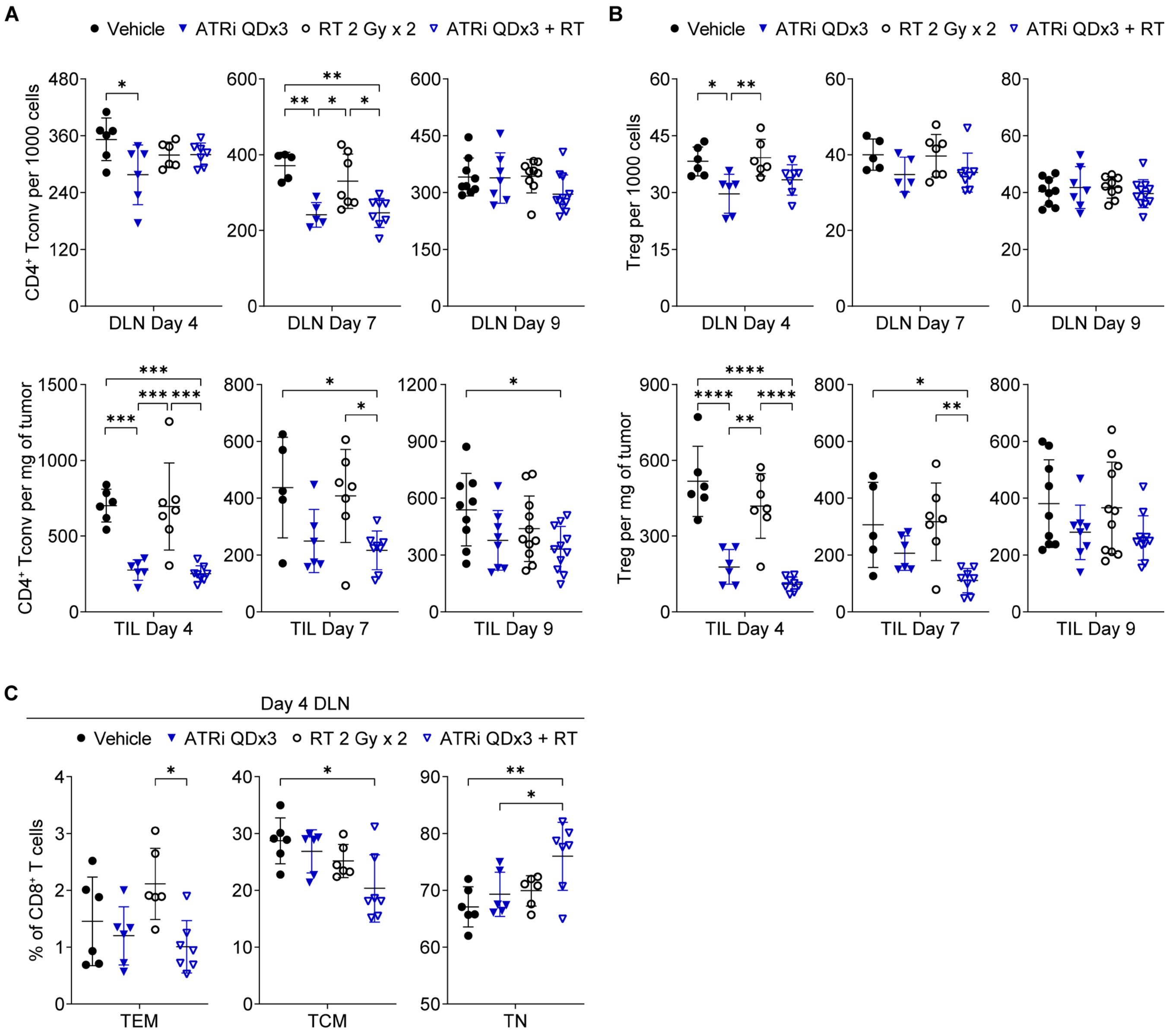

Figure S3. Short-course ATRi impacts T cells populations.

CT26 tumor-bearing mice were treated with ATRi QDx3, RT (2 Gy x 2), ATRi QDx3 + RT, or vehicle. DLN and TIL were immunoprofiled at Day 4, Day 7, and Day 9. **A-B.** Quantitation of $CD4^+$ Tconv (**A**) and Treg (**B**) in the DLN (per 1000 cells stained) and TIL (per mg of tumors). **C.** Quantitation of $CD8^+$ TEM, TCM, and TN, as percentages of total $CD8^+$ T cells, in the DLN at Day 4. **A-C.** Data from at least 3 (Day 4), 2 (Day 7), or 4 (Day 9) independent experiments with 1-4 mice per group. n at Day 4 = 6 Vehicle, 6 ATRi QDx3, 7 RT (6 DLN), 8 ATRi QDx3 + RT (7 DLN). n at Day 7 = 5 Vehicle, 6 ATRi QDx3 (5 DLN), 7 RT, 8 ATRi QDx3 + RT. n at Day 9 = 9 Vehicle, 8 ATRi QDx3 (7 DLN), 11 RT (9 DLN), 11 ATRi QDx3 + RT. Mean and SD bars shown. *p<0.05, **p<0.01, ***p<0.001, ****p<0.0001 by ANOVA with Tukey's multiple comparisons test.

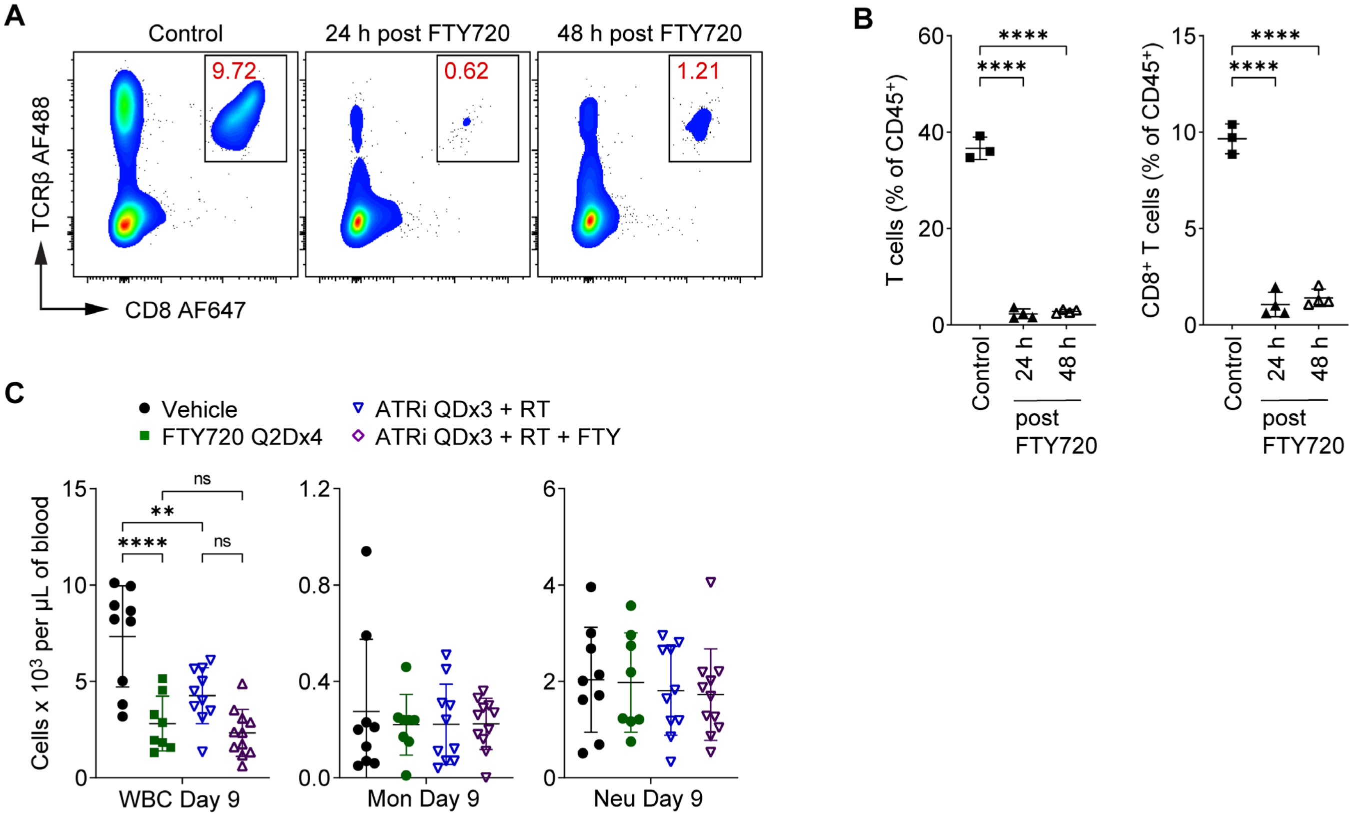

Figure S4. Validation of FTY720 dose and schedule for sequestration of lymphocytes in the peripheral lymphoid tissues.

A-B. BALB/c mice were injected with a single dose of 1 mg/kg FTY720, peripheral blood was collected at 24 h or 48 h post injection, and PBMCs were immunoprofiled. **A.** Representative cytograms depicting PBMCs expressing of TCRβ and CD8. **B.** Quantitation of circulating T cells (TCRβ^{+}) and CD8^{+} T cells (TCRβ^{+}CD8^{+}) as percentages of total CD45^{+} PBMCs. Data from one experiment with 3 control mice and 4 FTY720-treated mice at each time point. Mean and SD bars shown. ****$p<0.0001$ by ANOVA with Tukey's multiple comparisons test. **C.** CT26 tumor-bearing mice were treated with ATRi QDx3 + RT or vehicle, with or without 1 mg/kg FTY720 on days 1, 3, 5, and 7 (Q2Dx4). CBC were performed at Day 9 to quantitate white blood cells (WBC), monocytes (Mon), and neutrophils (Neu) per µL of blood. Data from 3 independent experiments with 2-4 mice per group. n = 9 Vehicle, 8 FTY720 Q2Dx4, 10 ATRi QDx3 + RT, 11 ATRi QDx3 + RT + FTY. Mean and SD bars shown. **$p<0.01$, ****$p<0.0001$, ns (not significant) by ANOVA with Sidak's multiple comparisons test, with brackets denoting the comparisons performed. Brackets not shown for Mon or Neu as no comparisons were significant.

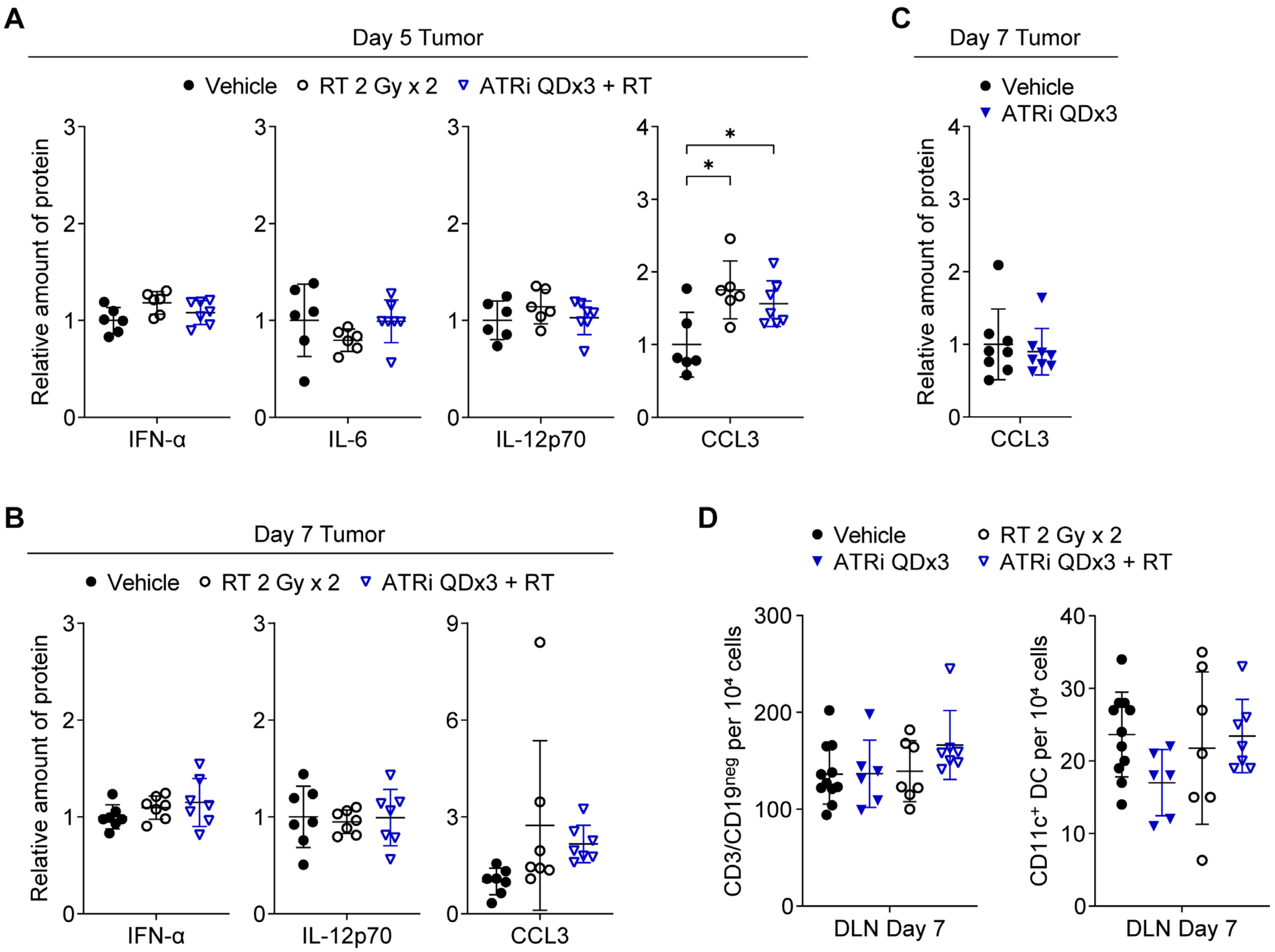

Figure S5. Impact of short-course ATRi and radiotherapy on proinflammatory cytokines and chemokines in the tumor microenvironment.

A-B. CT26 tumor-bearing mice were treated with RT 2 Gy x 2, ATRi QDx3 + RT, or vehicle. **A-B.** Quantitation of the relative amount of protein (normalized to vehicle control) for a subset of inflammatory cytokines and chemokines in tumors at (**A**) Day 5 and (**B**) Day 7. Day 5 data from one cohort of mice with n = 6 Vehicle, 6 RT, 7 ATRi QDx3 + RT. Day 7 data from two independent cohorts of mice, each with 3-4 mice per group, with total n = 7 Vehicle, 7 RT, 7 ATRi QDx3 + RT. **C.** CT26 tumor-bearing mice were treated with ATRi QDx3 or vehicle and the relative amount of CCL3 protein (normalized to vehicle control) in tumors at Day 7 was quantified. Data from 3 independent cohorts of mice, each with 2-3 mice per group, with total n = 8 mice per group. **D.** Quantitation (per 10^4 cells stained) of the relative number of $CD3^{neg}CD19^{neg}$ cells and $CD11c^+$ DC in DLN immunoprofiled at Day 7. Data from two independent experiments with 3-4 Vehicle, RT, and ATRi QDx3 + RT mice per group and one experiment with 3 Vehicle and 6 ATRi QDx3 mice per group. n = 11 Vehicle, 6 ATRi QDx3, 7 RT, 7 ATRi QDx3 + RT. **A-D**. Mean and SD bars shown. **A, B, D.** *$p<0.05$ by ANOVA with Tukey's multiple comparisons test.

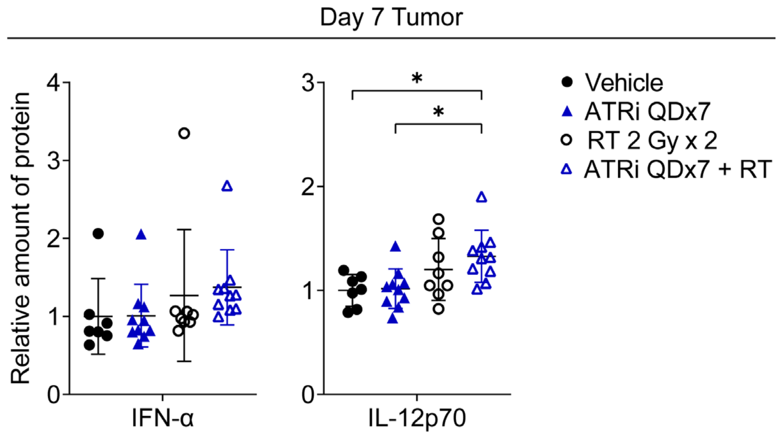

Figure S6. Impact of prolonged daily ATRi treatment on proinflammatory cytokines in the tumor microenvironment.

CT26 tumor-bearing mice were treated with ATRi on days 1-7 (ATRi QDx7), radiotherapy on days 1-2 (RT 2 Gy x 2), ATRi QDx7 + RT, or vehicle. Shown is the quantitation of the relative amounts (compared to vehicle control) of IFN-α and IL-12p70 protein in tumors at Day 7. Data from at least 4 independent cohorts of mice, each with 1-4 mice per group. n = 7 Vehicle, 10 ATRi QDx7, 8 RT, 10 ATRi QDx7 + RT. Mean and SD bars shown. *$p<0.05$ by ANOVA with Tukey's multiple comparisons test.

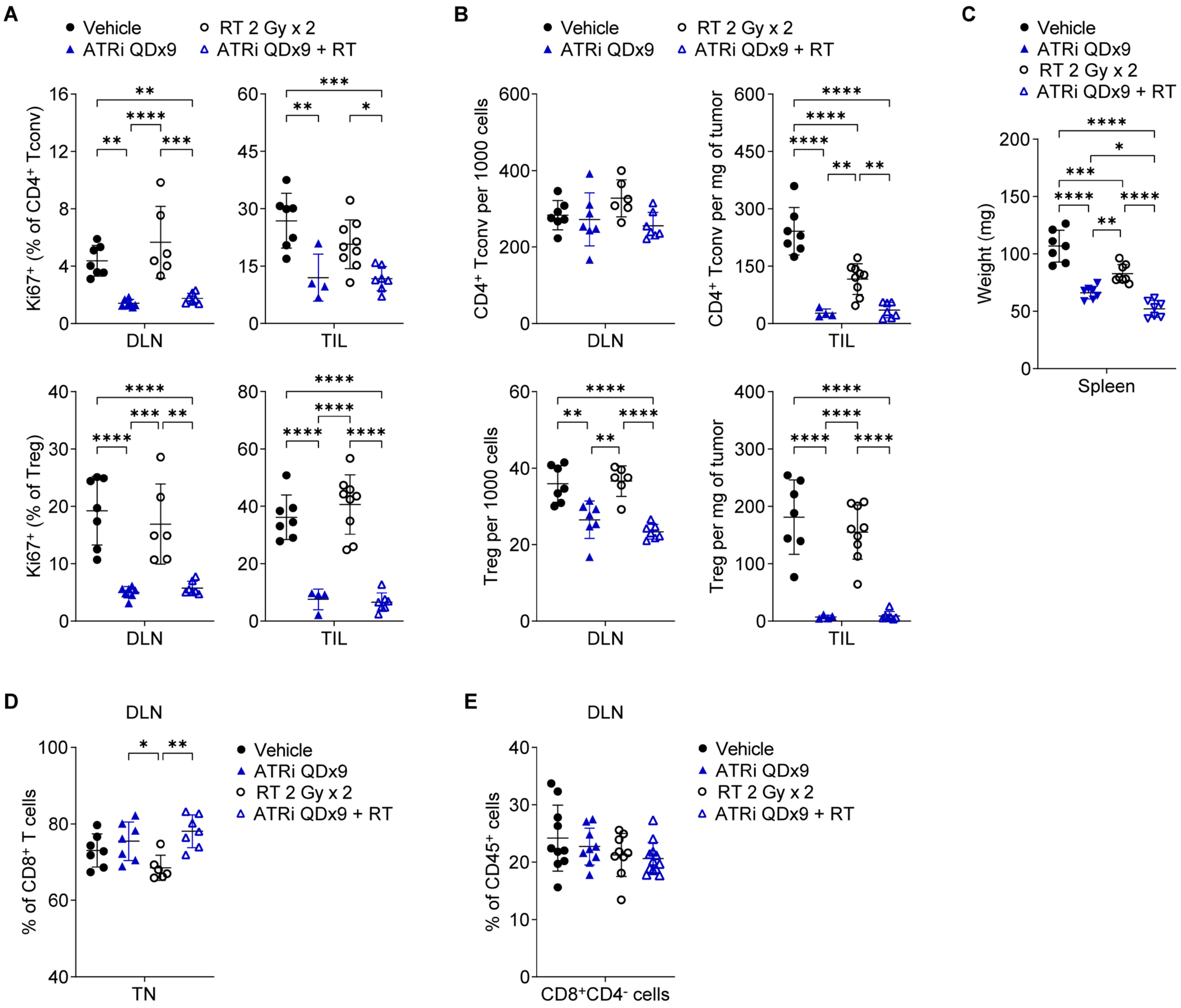

Figure S7. Prolonged daily ATRi treatment restrains the adaptive T cell response in the periphery. A-E. CT26 tumor-bearing mice were treated with ATRi on days 1-9 (ATRi QDx9), radiotherapy on days 1-2 (RT 2 Gy x 2), ATRi QDx9 + RT, or vehicle. DLN and TIL were immunoprofiled at day 9, 1 h after the final dose of ATRi. **A.** Quantitation of proliferating (Ki67^{+}) CD4^{+} Tconv and Treg, as percentages of the corresponding parent population, in DLN and TIL. **B.** Quantitation of the numbers of CD4^{+} Tconv and Treg in DLN (per 1000 cells stained) and TIL (per mg of tumor). **C.** Quantitation of whole spleens weights at Day 9. **A-C.** Data from two independent experiments (one for ATRi QDx9 TIL) with 2-5 mice per group. n = 7 Vehicle, 7 ATRi QDx9 DLN (4 TIL), 9 RT TIL (6 DLN), 7 ATRi QDx9 + RT. **D.** Quantitation of CD8^{+} TN, as a percentage of total CD8^{+} T cells, in the DLN. **E.** Quantitation of total CD8^{+} T cells, as a percentage of total CD45^{+} immune cells, in the DLN. **D-E.** Data from 5 independent experiments with 1-4 mice per group. n = 10 Vehicle, 9 ATRi QDx9, 9 RT, 12 ATRi QDx9 + RT. **A-E.** Mean and SD bars shown. *$p<0.05$, **$p<0.01$, ***$p<0.001$, ****$p<0.0001$ by ANOVA with Tukey's multiple comparisons test.

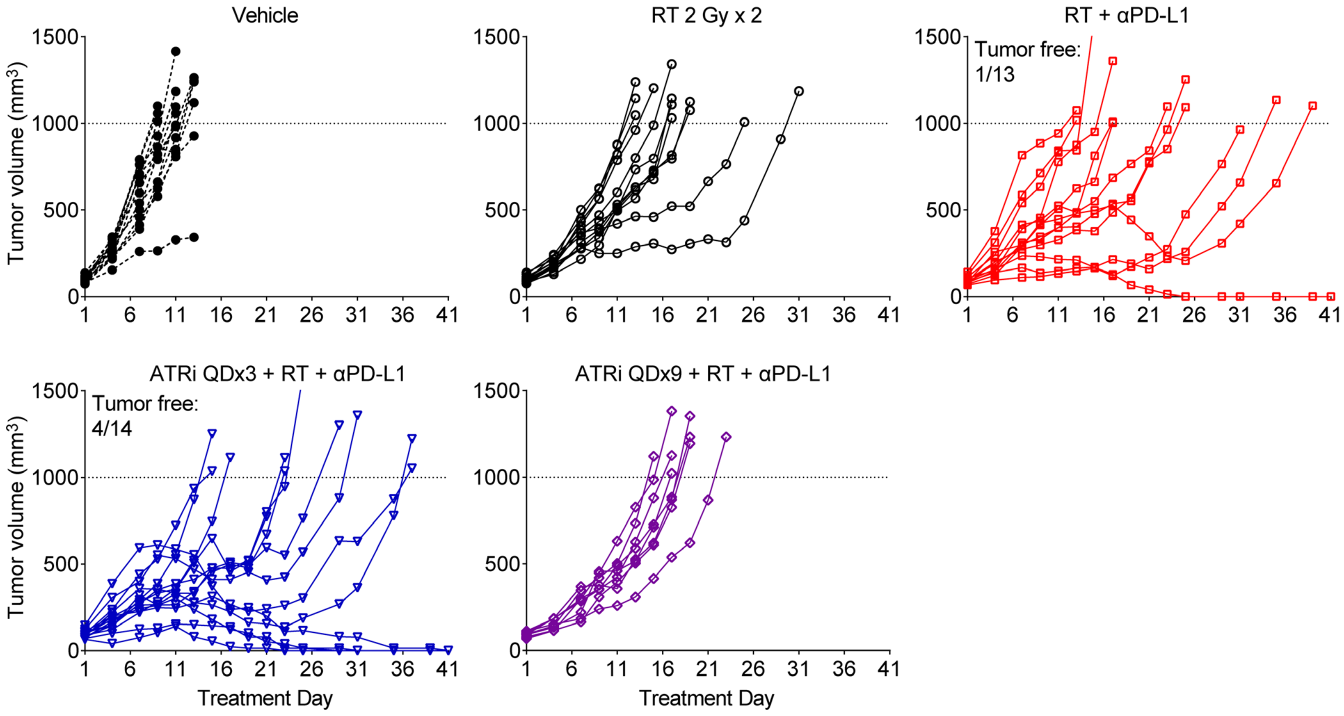

Figure S8. Cessation of ATRi treatment is necessary for extended survival after ATRi combined with sequential radiotherapy plus PD-L1 blockade

CT26 tumor-bearing mice were treated with radiotherapy on days 1-2 (RT 2 Gy x 2), RT + anti-PD-L1 antibody (αPD-L1, days 7, 9, 11), ATRi QDx3 + RT + αPD-L1, ATRi QDx9 + RT + αPD-L1, or vehicle. Individual growth curves depicting tumor volume over time are shown, and the number of tumor-free mice (complete responders), when present, are noted.

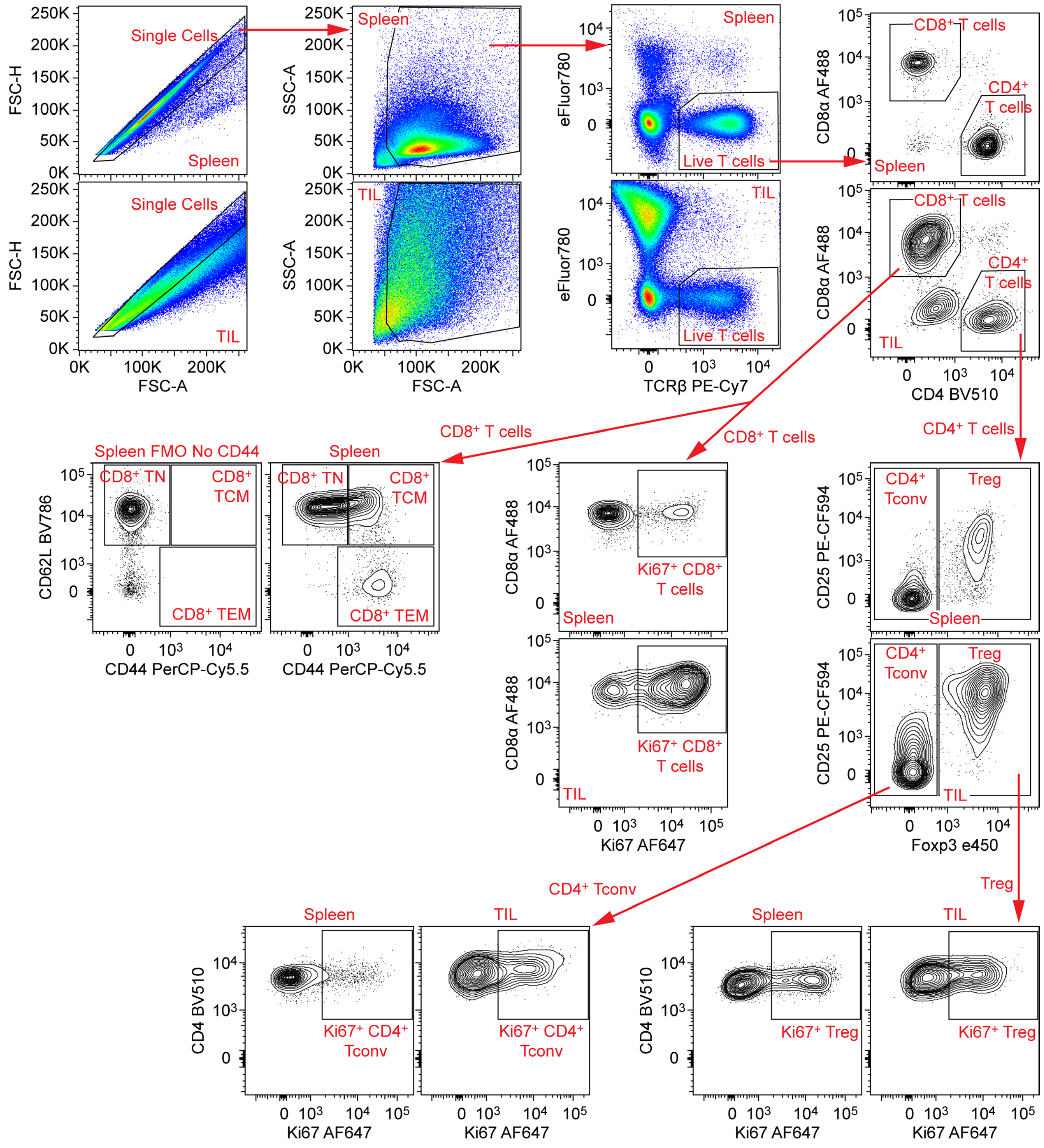

Figure S9. Flow cytometry gating strategy to identify T cells, proliferating T cells, and CD8^{+} T cell activation phenotypes in the TIL, DLN, and spleens.

Any unstable portions of the run were gated out prior to analysis. After single cell gating (FSC-A vs. FSC-H) to remove doublets/clumps and scatter gating (FSC-A vs. SSC-A) to exclude debris and, live T cells (efluor780^{neg} and TCRβ^{+}) were gated and then subset into CD8^{+} and CD4^{+} T cells. CD8^{+} T cells were examined for Ki67 expression to identify proliferating (Ki67^{+}) CD8^{+} T cells as well as expression of CD62L and CD44 to identify naïve (CD62L^{hi}CD44^{lo}), central memory (TCM, CD62L^{hi}CD44^{hi}), and

effector/effector memory (TEM, $CD62L^{lo}CD44^{hi}$) $CD8^+$ T cells. $CD4^+$ T cells were subset into conventional $CD4^+$ T cells (Tconv, $Foxp3^{neg}$) and regulatory T cells (Treg, $Foxp3^+$). CD25 was included to improve resolution between the $CD4^+$ T conv and Treg populations. Both the $CD4^+$ Tconv and Treg populations were then examined for Ki67 expression to identify proliferating ($Ki67^+$) cells in each pool. Spleen and TIL examples are shown for gating. A spleen fluorescence-minus-one control is shown for empiric determination of the $CD44^{hi}$ gate.

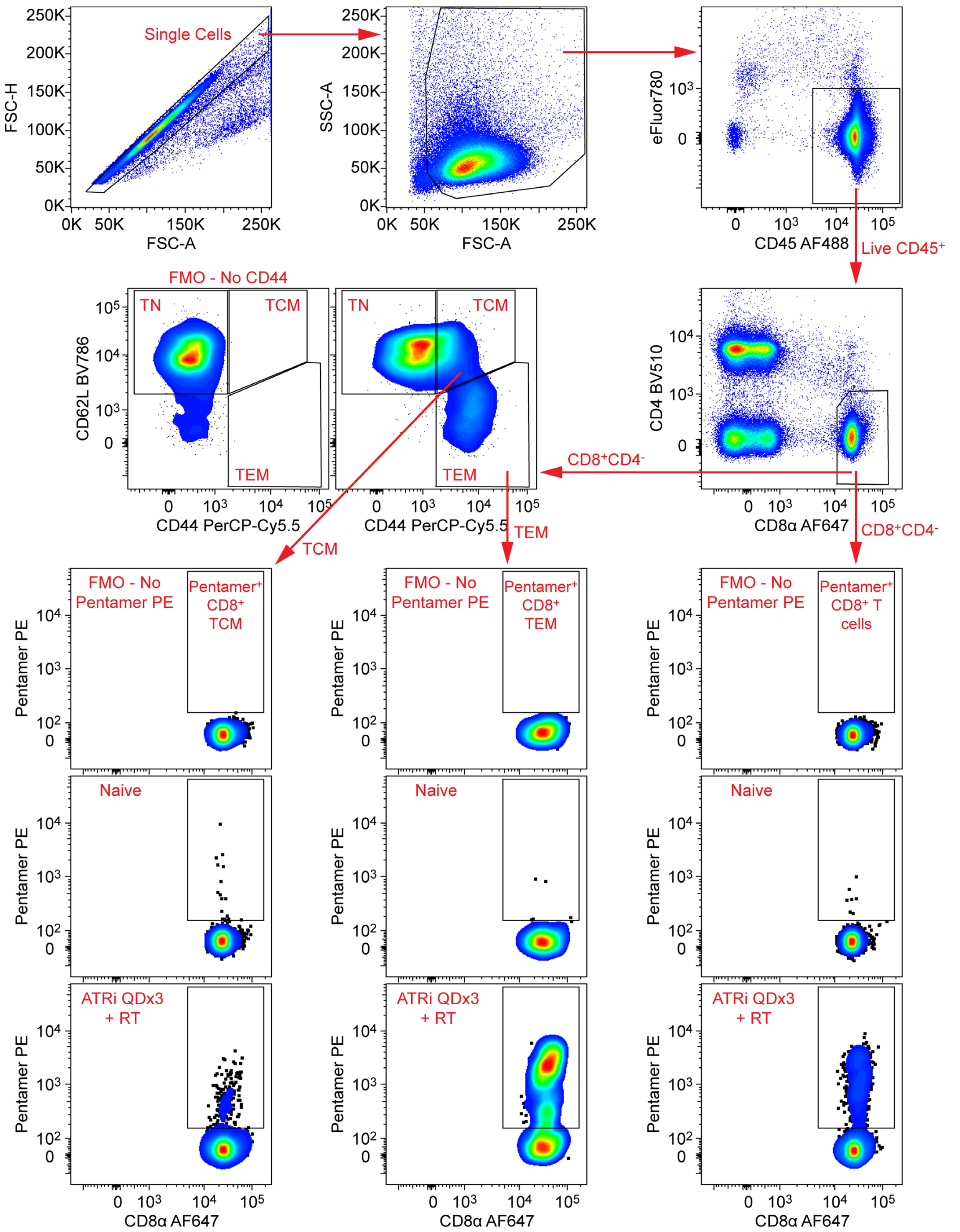

Figure S10. Flow cytometry gating strategy to identify tumor antigen specific CD8$^+$ T cells in the DLN.

Any unstable portions of the run were gated out prior to analysis. After single cell gating (FSC-A vs. FSC-H) to remove doublets/clumps and scatter gating (FSC-A vs. SSC-A) to exclude debris and, live immune cells (efluor780^{neg} and CD45^{+}) in the DLN were gated for further analysis. CD8^{+}CD4^{-} cells were examined for AH1 Pentamer binding to identify tumor antigen-specific (Pentamer^{+}) CD8^{+} T cells. In addition, CD8^{+}CD4^{-} cells were examined for expression of CD62L and CD44 to identify naïve (CD62L^{hi}CD44^{lo}), central memory (TCM, CD62L^{hi}CD44^{hi}), and effector/effector memory (TEM, CD62L^{lo}CD44^{hi}) CD8^{+} T cells. TCM and TEM were further examined for AH1 Pentamer binding to identify tumor antigen-specific CD8^{+} TCM and tumor antigen-specific CD8^{+} TEM. Fluorescence-minus-one controls are shown for empiric determination of CD44 and Pentamer gates. DLN from a naïve (no tumor) control mouse, included with each experiment, is shown to demonstrate low background Pentamer binding. DLN from an ATRi QDx3 plus RT-treated mouse is shown to demonstrate positive Pentamer binding.

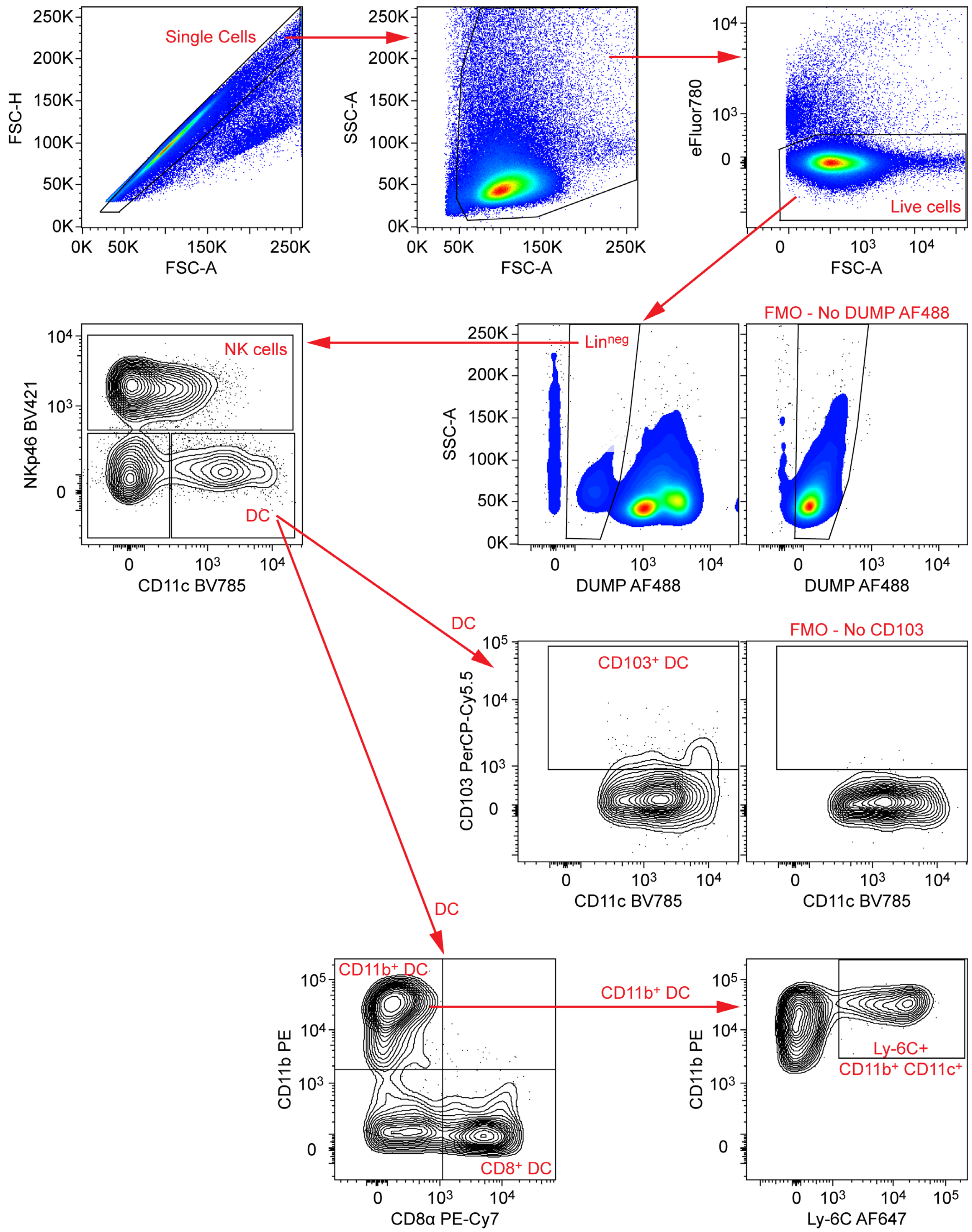

Figure S11. Flow cytometry gating strategy to identify innate immune cells in the DLN.

Any unstable portions of the run were gated out prior to analysis. After single cell gating (FSC-A vs. FSC-H) to remove doublets/clumps and scatter gating (FSC-A vs. SSC-A) to exclude debris, live cells (efluor780^{neg}) in the DLN were gated, and T and B cells (CD3^{+} or CD19^{+}, DUMP channel) were excluded. The Lin^{neg} (CD3^{neg}CD19^{neg}) cells were subset into NK cells (NKp46^{+}), dendritic cells (DC, CD11c^{+} NKp46^{neg}) and CD11c^{neg}NKp46^{neg}. DC were further profiled for CD103, CD8α, or CD11b expression to identify CD103^{+} DC, CD8^{+} DC, or CD11b^{+} DC, respectively. Ly-6C expression on CD11b^{+} DC was also examined. FMO control plots are shown for DUMP and CD103.

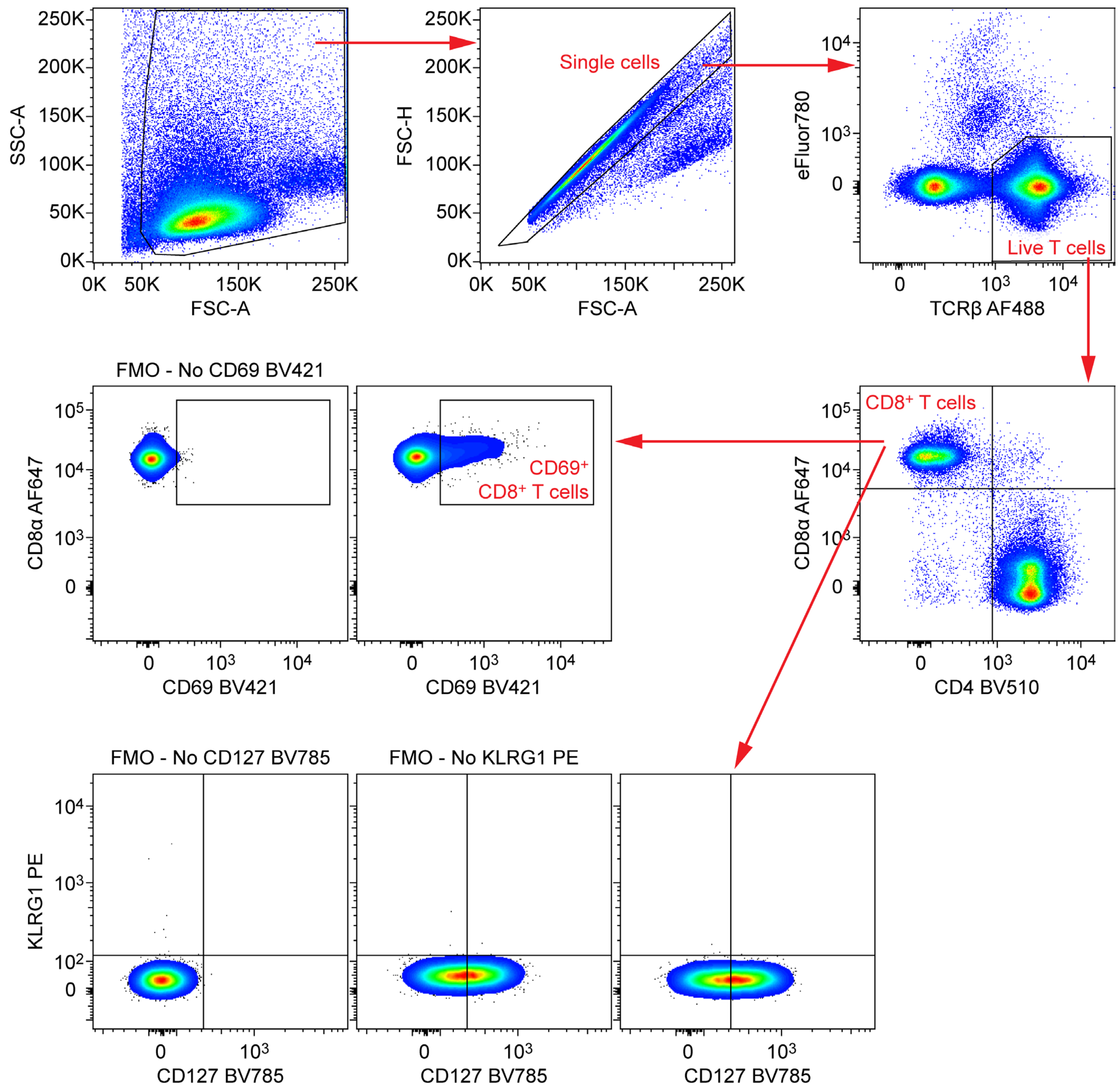

Figure S12. Flow cytometry gating strategy to identify newly activated CD8$^+$ T cells in the DLN. Any unstable portions of the run were gated out prior to analysis. After scatter gating (FSC-A vs. SSC-A) to exclude debris and single cell gating (FSC-A vs. FSC-H) to remove doublets/clumps, live T cells (efluor780^{neg} and TCR$^+$) in the DLN were gated and subset into CD8$^+$ T cells and CD4$^+$ T cells. CD8$^+$ T cells were further examined for expression of CD69 to identify newly activated CD8$^+$ T cells in the DLN. KLRG1 PE and CD127 BV785 were included in the staining panel, but CD8$^+$ T cells expressing these markers were not reported due to very low abundance of KLRG1$^+$ CD8$^+$ T cells and weak staining of CD127 on CD8$^+$ T cells. FMO control plots are shown for CD69, KLRG1, and CD127.

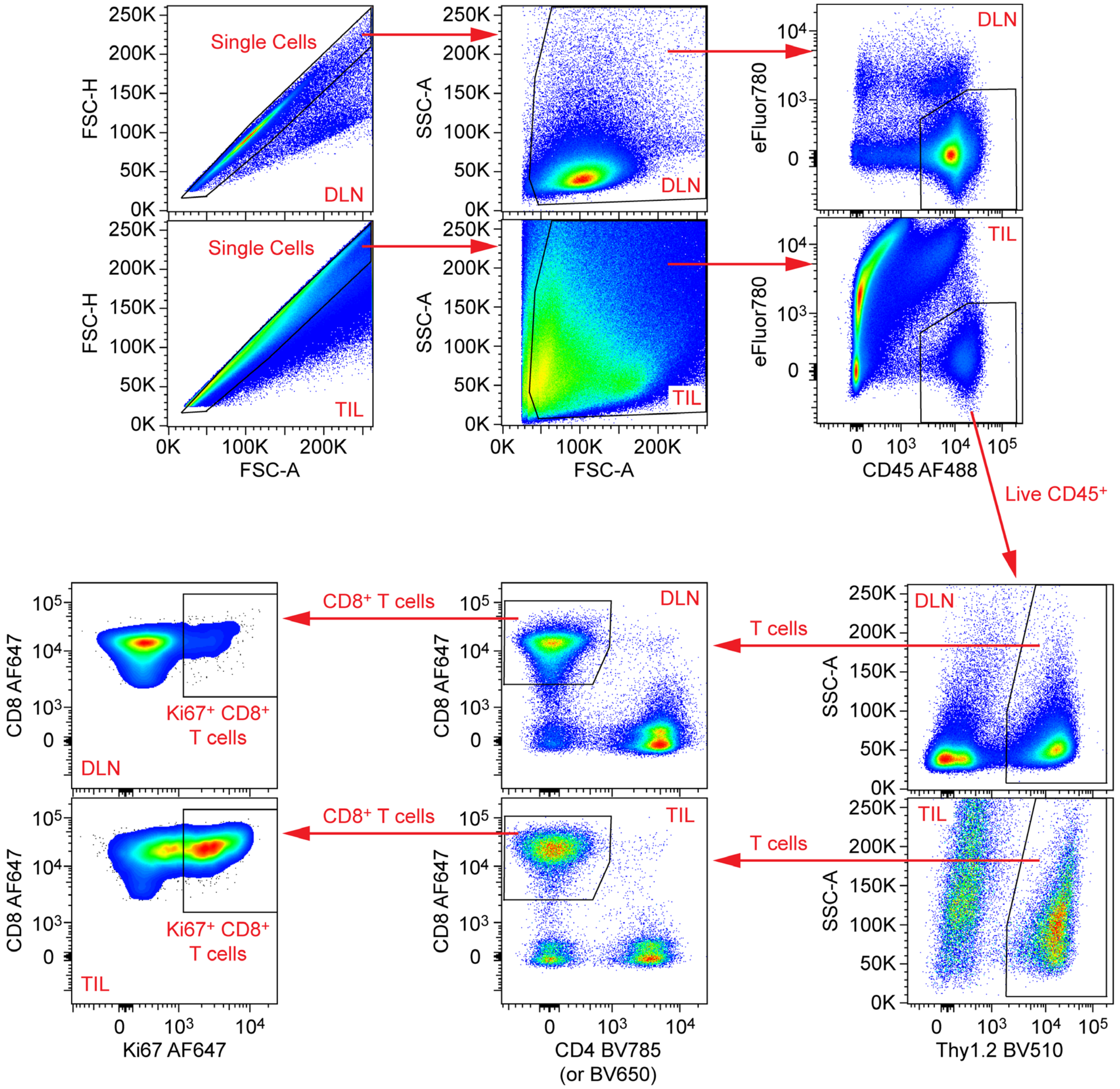

Figure S13. Flow cytometry gating strategy to identify proliferating CD8$^+$ T cells in the TIL and DLN of B16 tumor-bearing mice.

Any unstable portions of the run were gated out prior to analysis. After single cell gating (FSC-A vs. FSC-H) to remove doublets/clumps and scatter gating (FSC-A vs. SSC-A) to exclude debris and, live CD45 immune cells (efluor780^{neg} and CD45$^+$) were gated were gated for further analysis. T cells were gated based on Thy1.2 (CD90.2) expression and subset into CD8$^+$ T cells and CD4$^+$ T cells. CD8$^+$ T cells were examined for Ki67 expression to identify proliferating (Ki67$^+$) CD8$^+$ T cells in the TIL and DLN.

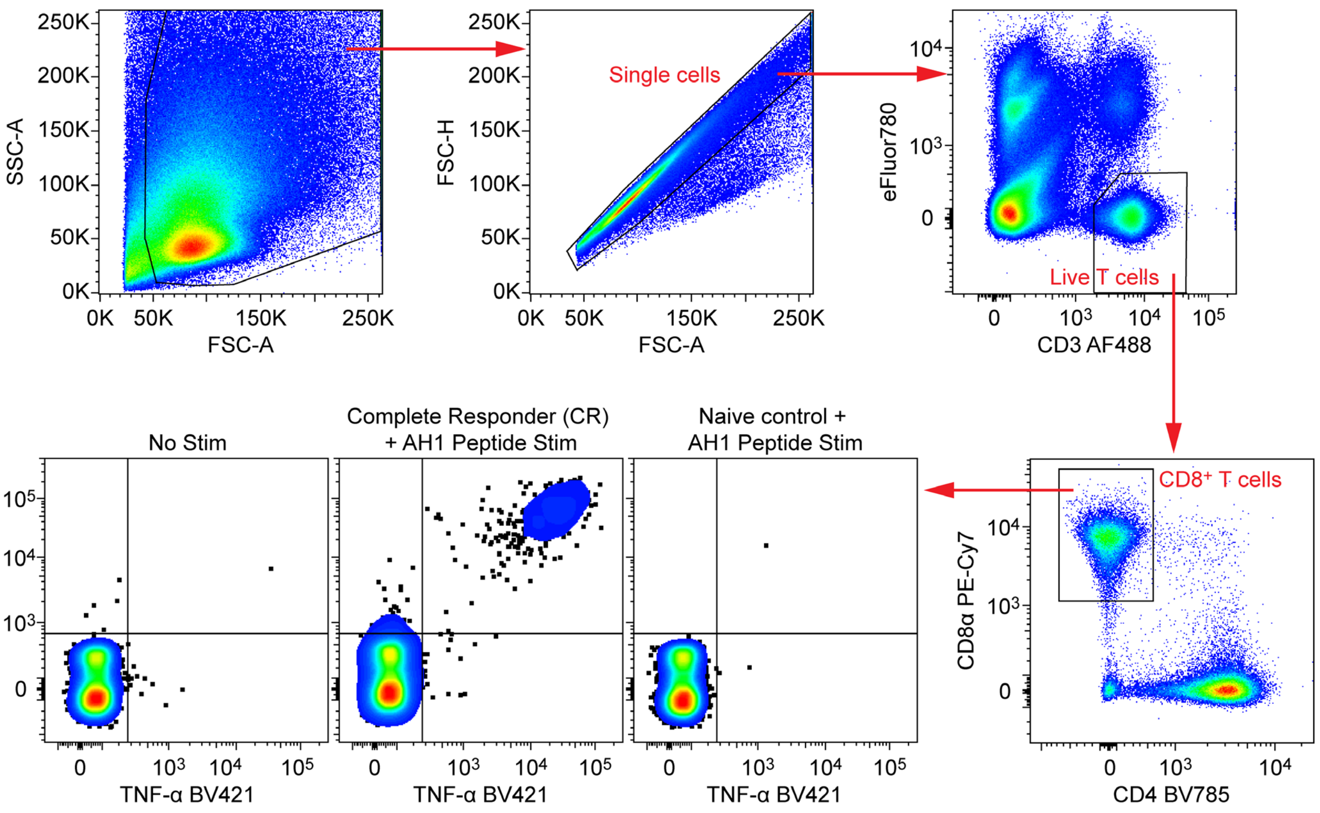

Figure S14. Flow cytometry gating strategy to identify cytokine-producing CD8^{+} T cells following stimulation of complete responder BALB/c splenocytes with the CT26 tumor antigen peptide AH1. Any unstable portions of the run were gated out prior to analysis. After scatter gating (FSC-A vs. SSC-A) to exclude debris and single cell gating (FSC-A vs. FSC-H) to remove doublets/clumps, live T cells (efluor780^{neg} and CD3^{+}) were gated and subset into CD8^{+} T cells and CD4^{+} T cells. CD8^{+} T cells were examined for expression of IFN-γ and TNF-α, and representative plots are shown for unstimulated (No Stim) complete responder, AH1 peptide-stimulated complete responder, and AH1 peptide-stimulated naïve control samples.
